# Construction and Validation of a Contextualized Competency Framework for Newly Recruited Nurses in Maternal and Child Health Hospitals

**DOI:** 10.3390/healthcare14121772

**Published:** 2026-06-19

**Authors:** Nan Wan, Siying Liu, Zhaoyi Ye, Yongqi He, Yutao Lan

**Affiliations:** Department of Nursing, Guangdong Pharmaceutical University, Jianghai Street No. 283, Guangzhou 510310, China; 2112376034@stu.gdpu.edu.cn (N.W.); 2112376044@stu.gdpu.edu.cn (S.L.); 2112276021@stu.gdpu.edu.cn (Z.Y.); 2112376019@stu.gdpu.edu.cn (Y.H.)

**Keywords:** maternal–child health services, nurses, clinical competence, psychometrics, self-assessment

## Abstract

**Objectives:** Existing nursing competency instruments are generally designed for broad nursing populations and may not fully capture the observable, task-linked, and developmental requirements of newly recruited nurses in maternal and child health (MCH) hospitals. This study developed the Newly recruited nurses’ Competency Framework for Maternal and Child Health Hospitals (NCF-MCH) and preliminarily validated its corresponding self-assessment tool. **Methods:** Guided by Benner’s novice-to-expert perspective and Mills’ reconceptualised competency terminology, the framework was developed through a structured literature and framework review, item mapping, two-round Delphi consultation, and pilot testing. A cross-sectional survey was conducted among newly recruited nurses from four MCH hospitals. Exploratory factor analysis, confirmatory factor analysis, and reliability and validity analyses were performed. **Results:** The final framework included six domains and 70 items. The scale showed high internal consistency (Cronbach’s α = 0.944), but weak total split-half reliability (0.585). Exploratory factor analysis using principal axis factoring with Promax rotation suggested a six-factor solution explaining 59.01% of the variance. Confirmatory factor analysis showed acceptable absolute fit (χ^2^/df = 1.675, SRMR = 0.0595, RMSEA = 0.055), whereas incremental fit was marginal (TLI = 0.861, CFI = 0.866). Convergent and discriminant validity analyses provided preliminary support for the multidimensional structure. **Conclusions:** The NCF-MCH provides a context-specific framework and corresponding self-assessment tool for describing self-perceived competency among newly recruited nurses in MCH hospitals. It may inform professional transition programs and competency-based training, but further external, longitudinal, and objective validation is required.

## 1. Introduction

With changes in fertility policy, increasing demand for maternal and child health services, and the expansion of nursing care from disease-centered care to life-course health management, competency requirements for nurses in maternal and child health (MCH) hospitals have become increasingly comprehensive, specialized, and context-specific [1,2]. Compared with general hospitals, MCH nursing involves not only clinical care in maternal, neonatal, pediatric, and women’s health settings, but also health education, breastfeeding support, family-centered care, psychological support, follow-up management, and early risk identification [3,4]. Newly recruited nurses in these settings are expected to integrate assessment, communication, education, collaboration, and risk-response abilities in highly specific MCH scenarios [5,6].

The transition period for newly graduated and early-career nurses is often characterized by transition shock, insufficient confidence in clinical judgment, difficulty prioritizing care, limited interprofessional communication experience, and uncertainty in responding to complex clinical situations [7,8]. These challenges may be intensified in MCH hospitals because newly recruited nurses must care for women, neonates, children, and families while recognizing early risks and communicating timely concerns to senior nurses or multidisciplinary team members. Existing standardized training and competency assessment tools remain useful for assessing general nursing competence, such as clinical care, communication, professionalism, ethics, and continuing development. However, these broad domains do not always indicate whether early-career nurses can perform MCH-specific tasks across maternal, neonatal, pediatric, family education, and early risk-escalation contexts. This mismatch may limit the ability of nurse educators and managers to identify specific training needs and monitor early role adaptation in MCH hospital settings.

Competency frameworks have been widely used in health workforce development, performance evaluation, and educational design [9]. Existing nursing competency frameworks and inventories have made important contributions by identifying broad domains such as clinical care, patient safety, communication, professionalism, ethical practice, teamwork, leadership, continuing education, and evidence-based practice [10]. However, many frameworks remain at a relatively broad level and give less attention to how competencies are enacted as observable behaviours, practice activities, and specific tasks in real clinical settings [9,11]. For newly recruited nurses in MCH hospitals, three issues remain insufficiently addressed: how broad competency domains should be translated into MCH-specific nursing tasks; what level of performance is developmentally appropriate within the first two years of employment; and how areas such as health advocacy, leadership, quality improvement, and research should be framed as foundational or participatory behaviours rather than independent expert-level responsibilities.

To strengthen practice orientation, competency development in global health has increasingly shifted from abstract attribute-based models toward task-linked and context-based approaches [12]. Building on the reconceptualization of competency framework terminology proposed by Mills and colleagues [11], the World Health Organization developed a structured framework for universal health coverage based on domains, competencies, behaviours, practice activities, and tasks [13]. Guided by this logic, the present study distinguished the conceptual framework from the measurement scale. The competency framework was used to organize competency requirements for newly recruited nurses in MCH hospitals, whereas the corresponding self-assessment scale operationalized the framework by using task- and behaviour-level indicators as measurable items.

Competency among newly recruited nurses is shaped not only by clinical context but also by developmental stage. Benner’s novice-to-expert theory emphasizes that nursing competence develops through experience and clinical practice [14]. In this study, Benner’s theory was not used to construct a full five-stage developmental scale. Rather, it was used as a developmental lens to define the expected proficiency boundary of the target population and to avoid expert-level item wording. Because the target population was newly recruited nurses within two years of employment, items were intended to describe foundational, assisted, and participatory behaviours, such as recognizing risks, reporting concerns, collaborating with senior nurses and multidisciplinary team members, participating in quality improvement, and applying evidence under guidance [15].

The contribution of this study lies in translating general nursing competency concepts into MCH-specific, task-linked, and developmentally appropriate self-assessment items for newly recruited nurses. Therefore, this study aimed to develop the Newly Recruited Nurses’ Competency Framework for Maternal and Child Health Hospitals (NCF-MCH) and preliminarily validate its corresponding self-assessment tool. Specifically, the study sought to identify core competency domains relevant to MCH nursing practice, operationalize these domains into observable task- and behaviour-level items, and examine the initial psychometric evidence of the tool. The NCF-MCH is intended to provide an initial reference for describing self-perceived competency and identifying perceived training needs, while further external, longitudinal, and objective validation remains necessary.

## 2. Materials and Methods

This study was a methodological study involving the development and preliminary psychometric validation of a self-assessment tool derived from a context-specific competency framework. The study consisted of two stages: framework development and psychometric validation. The framework development stage included a structured literature and framework review, item extraction and mapping, two-round Delphi expert consultation, and pilot testing. The validation stage used a cross-sectional survey to examine item performance, content validity, construct validity, convergent and discriminant validity, reliability, and test–retest reliability. The reporting and interpretation of measurement properties were informed by the COSMIN recommendations for studies on measurement properties [16].

### 2.1. Development Stage

The development stage was guided by Benner’s theoretical perspective, Mills’ reconceptualised competency terminology, and contextualized competency framework adaptation logic [14,17]. Existing competency frameworks were reviewed [13,18,19], mapped, adapted, and refined according to the intended population, MCH practice context, and expected proficiency level.

#### 2.1.1. Structured Literature and Framework Review

A structured literature and framework review was conducted to identify relevant evidence on nursing competency, newly recruited nurses, maternal and child health nursing, and competency framework development. The following electronic databases were searched: PubMed, Web of Science, CINAHL, and CNKI. In addition, official websites of international and national health and nursing organizations, including the World Health Organization, were manually searched. Reference lists of relevant articles and framework documents were also screened.

Search terms combined competency-related, population-related, and MCH-context-related terms. An example English search strategy was: (“competency framework” OR “clinical competence” OR “nursing competency” OR “professional competence”) AND (“new nurses” OR “newly recruited nurses” OR “newly graduated nurses”) AND (“maternal and child health” OR “maternal nursing” OR “neonatal nursing” OR “pediatric nursing”). Equivalent Chinese terms were used in CNKI.

Documents were included if they described nursing or health workforce competency domains, competency statements, behaviours, practice activities, tasks, knowledge, skills, or role expectations relevant to newly recruited nurses or MCH-related clinical contexts. Documents unrelated to nursing or health workforce competency, documents without extractable competency-related information, duplicate publications, commentaries without clear competency content, documents focused exclusively on undergraduate students or pre-licensure education without relevance to newly recruited nurses’ post-entry practice, and documents without available full text or sufficient framework details were excluded.

All records were imported into EndNote, and duplicates were removed. Two researchers independently screened titles, abstracts, and full texts. Disagreements were resolved through team discussion. The search identified 1940 records; 1531 were excluded after duplicate removal and title/abstract screening. Forty-nine full-text documents were assessed for eligibility, and 23 literature and framework documents were finally included for framework development and item generation.

#### 2.1.2. Item Extraction, Mapping, and Contextual Adaptation

A standardized extraction form was used to collect information on source, target population, clinical context, competency domain, competency statement, behaviour, practice activity, task, knowledge or skill component, and relevance to newly recruited nurses in MCH hospitals. The included documents were classified according to their role in framework development. Direct item-content sources provided MCH-related nursing, maternal–newborn, pediatric, neonatal, or newly recruited nurse competency content. General nursing competency sources provided broader domains such as clinical care, communication, professionalism, leadership, learning, and evidence-based practice. Conceptual or structural framework sources, including WHO competency frameworks, were used to guide the organization of domains, competencies, behaviours, practice activities, and tasks rather than to provide MCH-specific item content directly. A full list of the 23 included documents and their role in item generation is provided in Supplementary Table S1.

Mills’ terminology was used as an organizing logic for transforming broad competency concepts into observable, assessable, task- or behaviour-level item wording. Broad competency statements were not used directly as questionnaire items. Instead, they were linked to MCH-related practice activities, such as maternal assessment, neonatal observation, breastfeeding support, family education, infection prevention, risk reporting, interprofessional communication, and quality improvement participation, and were then rewritten as self-assessment items [20].

Benner’s novice-to-expert theory was used to define the expected proficiency boundary. Because the target population was newly recruited nurses within two years of employment, item wording emphasized foundational and participatory behaviours, such as recognizing risks, reporting concerns, communicating with women and families, collaborating with senior nurses and multidisciplinary team members, participating in quality improvement, and applying evidence under guidance. Expert-level expectations, such as independently leading complex clinical decisions, managing departments, or conducting independent research, were avoided.

The initial item pool was developed by a research team of four researchers. Items were reviewed for conceptual clarity, developmental appropriateness, relevance to MCH practice, observability, and alignment with the framework structure. Items were revised, merged, or deleted when they were redundant, too broad, insufficiently observable, or beyond the expected role boundary of newly recruited nurses. This process generated an initial pool of 107 tertiary indicators. Supplementary Table S2 presents the primary source basis and contextual operationalisation process for the final items. Because multiple documents often supported overlapping competency concepts, Supplementary Table S2 reports the most directly relevant or representative sources for each item rather than exhaustively listing every document that informed the initial item pool.

#### 2.1.3. Delphi Consultation and Content Validity Assessment

Following the structured item extraction and contextual adaptation process, a two-round Delphi technique was conducted to further evaluate the relevance, importance, clarity, and developmental appropriateness of the preliminary items. The Delphi process was used to refine, rather than solely generate, the item pool.

A two-round Delphi technique was first conducted to screen and refine the preliminary items of the scale. The expert selection criteria included: (1) engagement in clinical practice or research related to maternal and child healthcare nursing, nursing management, or nursing education; (2) possession of ≥10 years of professional experience; (3) holding a bachelor’s degree or higher; and (4) agreement to participate in both rounds of the Delphi study. The Delphi panel mainly included senior nurses and nursing managers because this stage aimed to evaluate professional relevance, content coverage, and contextual appropriateness. The perspectives of newly recruited nurses were incorporated through the subsequent pilot test.

Experts completed the Delphi questionnaires independently and anonymously in each round. After the first round, group-level statistical results and anonymized qualitative comments were summarized and provided as controlled feedback before the second round. Expert authority was assessed using the authority coefficient. Consensus stability was evaluated using response rates, revision suggestions, changes in Kendall’s W, changes in item-level ratings, and whether new substantive comments emerged in the second round. Items were retained, revised, merged, or removed based on pre-specified criteria, including mean importance score ≥4.0, coefficient of variation ≤0.25, full-score rate >20%, content validity results, expert comments, theoretical relevance, MCH contextual appropriateness, and developmental suitability.

Content validity assessment was treated as methodologically distinct from the Delphi consensus process. Experts rated item relevance using a 4-point scale. The item-level content validity index (I-CVI) was calculated as the proportion of experts rating an item as 3 or 4, and the S-CVI/Ave was calculated as the average of all I-CVI values. Items were considered acceptable when I-CVI was >0.78 and S-CVI/Ave was >0.90 [21]. Expert comments were used to supplement the CVI results by identifying unclear wording, redundant content, missing domains, and concerns about construct representativeness [22].

#### 2.1.4. Pilot Test

A pilot test was conducted among newly recruited nurses within two years of employment from one MCH hospital. Participants completed the preliminary scale and provided feedback on item clarity, readability, relevance to actual work, and appropriateness for self-assessment. The pilot feedback was not analysed as a formal qualitative study; rather, it was used as a structured pretest to identify problems in comprehension, wording, and role appropriateness. Items identified as difficult to understand, overly abstract, too dense, or beyond the typical role experience of newly recruited nurses were revised by simplifying wording, adding behavioural specificity, replacing abstract expressions with task-based wording, or reframing advanced expectations as participatory behaviours. Major revisions are summarized in Supplementary Table S3.

### 2.2. Validation Stage

#### 2.2.1. Design and Participants

A cross-sectional study was conducted between September 2025 and December 2025 in four MCH hospitals in Eastern China. Eligible participants were newly recruited nurses within two years of employment, calculated from the date of signing the labor contract, who entered the position as fresh graduates without prior clinical nursing work experience and agreed to participate. Nurses who withdrew midway were excluded. A total of 554 participants were recruited [23].

#### 2.2.2. Instruments and Data Collection

The preliminary NCF-MCH included 70 items scored on a 5-point Likert scale, ranging from 1 = “Completely Incompetent” to 5 = “Fully Competent.” Higher scores indicated higher self-perceived competency in performing MCH-related tasks. A self-designed general information questionnaire was used to collect demographic and training-related information, including age, length of nursing practice, gender, educational background, and whether participants had completed rotations in MCH-related departments during internship.

Participants were recruited with the assistance of hospital nursing management departments. The study purpose and procedures were explained before data collection. Before completing the online questionnaire, participants were presented with an online informed consent form explaining the voluntary nature of participation, anonymity, confidentiality, and their right to withdraw at any time. Ethical approval was obtained from the Ethics Committee of Guangdong Provincial Maternal and Child Health Hospital (Approval No. 20250108).

#### 2.2.3. Statistical Analysis

Data were analysed using SPSS 26.0 and Amos 26.0. Demographic variables were described using frequencies, percentages, means, and standard deviations as appropriate. Item analysis included upper–lower 27% group comparisons, item-total correlations, corrected item-total correlations, and Cronbach’s α if item deleted. Item-retention decisions integrated statistical performance, theoretical relevance, MCH contextual importance, and Delphi expert feedback.

Internal consistency was assessed using Cronbach’s α. Split-half reliability was assessed by comparing odd- and even-numbered items. Test–retest reliability was assessed using the intraclass correlation coefficient (ICC) among 30 participants who completed the scale again after two weeks [24].

The total sample was randomly divided into two subsets using SPSS, with one subset used for EFA (n = 328) and the other for CFA (n = 226) [25,26]. EFA was conducted using principal axis factoring with Promax oblique rotation because the study aimed to explore latent competency constructs and the competency domains were theoretically expected to be related. Factor retention was based on eigenvalues ≥ 1.0, scree plot inspection, and factor interpretability. Items with communalities below 0.30, factor loadings below 0.50, or substantial cross-loadings were considered for exclusion [27,28].

CFA was conducted using maximum likelihood estimation. Model fit was evaluated using χ^2^/df, SRMR, RMSEA, TLI, and CFI. Values of χ^2^/df < 5, SRMR < 0.08, and RMSEA < 0.08 were considered acceptable. CFI and TLI were interpreted together with overall model characteristics because incremental fit indices may be influenced by model complexity and sample size [29,30,31]. Composite reliability (CR) and average variance extracted (AVE) were calculated to assess convergent validity. Discriminant validity was evaluated using the Fornell–Larcker criterion. The significance level was set at *p* < 0.05 [32].

## 3. Results

### 3.1. Development Stage

#### 3.1.1. Item Pool Development and Content Validity

The demographic characteristics of the Delphi experts are shown in Table 1. Because age and years of professional experience were collected as categorical ranges in the Delphi questionnaire, they are reported as frequency and percentage rather than mean ± standard deviation. The expert panel included professionals from MCH nursing clinical practice, nursing education, and nursing management. All experts had at least 10 years of professional experience, and most held intermediate-level or senior professional titles. These characteristics supported the relevance of the panel for expert-informed content refinement, although the panel composition is further discussed as a limitation.

The first round of the Delphi method achieved a 100% expert response rate, with 15 experts providing suggestions for item refinement, including deletions, modifications, and consolidations. The second round achieved a 95.7% response rate, with no additional suggestions emerging during this round. After two rounds of Delphi consultation, the NCF-MCH was refined into 6 first-level indicators, 35 second-level indicators, and 70 third-level indicators. The 70 third-level indicators were used as the measurement items in the validation study. The authority coefficients for the two rounds were 0.875 and 0.900, suggesting a high level of expert authority. Kendall’s W increased from 0.352 in the first round to 0.405 in the second round, and both values were statistically significant, indicating improved but modest expert consensus [33].

Content validity was assessed using the Content Validity Index (CVI). The item-level CVI (I-CVI) ranged from 0.87 to 1.000, and the scale-level CVI (S-CVI) reached 0.94, indicating strong content validity for the NCF-MCH. The item-level I-CVI values are reported in Supplementary Table S2.

#### 3.1.2. Pilot Test

A pilot study was conducted with 30 newly recruited nurses from a maternal and child health hospital. The participants had an average age of 24.1 ± 1.09 years and an average work experience of 18.7 ± 7.21 months. Females accounted for 90% (n = 27) of the participants, while males made up 10% (n = 3). In terms of educational background, 33.3% (n = 10) held an associate degree, and 66.7% (n = 20) held a bachelor’s degree. All participants completed the scale within 20 min. Overall, most items were considered understandable. However, some items were perceived as abstract, dense, or beyond the typical experience of newly recruited nurses, especially those related to management, research, and advocacy. These items were revised to improve clarity, behavioural specificity, and role appropriateness. Major revisions are summarized in Supplementary Table S3.

### 3.2. Validation Stage

#### 3.2.1. Social and Demographic Characteristics of Participants

A total of 554 valid questionnaires were included. Using the random case selection function in SPSS, 328 cases were allocated to item analysis and EFA, and 226 cases were allocated to CFA. The demographic characteristics of the participants are shown in Table 2.

#### 3.2.2. Item Analysis

The item-level analysis results are shown in Supplementary Table S4. All items showed statistically significant discrimination between the high-score and low-score groups. The item-total correlations ranged from 0.261 to 0.674, and the corrected item-total correlations ranged from 0.233 to 0.656. Cronbach’s α if item deleted ranged from 0.942 to 0.944, indicating that deleting any single item did not substantially improve the overall internal consistency. However, several items showed corrected item-total correlations below 0.30, including Q1, Q3, Q4, Q40, Q52, Q53, Q62, Q63, and Q64. These items were retained at this stage because they represented theoretically and contextually important aspects of the framework, particularly health advocacy, management and leadership, and research-related competencies. Therefore, item retention was based on both statistical results and content relevance, and the current 70-item version should be regarded as an initial comprehensive framework requiring further refinement.

#### 3.2.3. Construct Validity

(1) Exploratory factor analysis

The total data (n = 554) were randomly split into two subsets. EFA was conducted on the first subset of 328 samples, encompassing all 70 items. Principal axis factoring with Promax oblique rotation was used to explore the latent factor structure. This approach was selected because the study aimed to identify underlying competency constructs and because the domains were theoretically expected to be related [23].

The correlation matrix verified an adequate sample size (Kaiser–Meyer–Olkin measure was 0.901), and the Bartlett test (χ^2^ = 17,992.840, *p* < 0.001) rejected the zero-correlations hypothesis. Following Kaiser’s criterion, principal axis factoring with Promax rotation suggested a six-factor solution, which explained 59.01% of the total variance. The first factor did not account for a dominant proportion of variance. However, this result should not be interpreted as ruling out common method bias, because all data were collected from the same respondents using a self-report format. The factor loadings for the scale items ranged from 0.64 to 0.856, all exceeding the threshold of 0.50. Each item’s communality value was above 0.387, surpassing the acceptable minimum of 0.30, with no items loading on multiple factors. The combination of the scree plot results, the Kaiser criterion (eigenvalue) and the factor meaningfulness led to the identification of a six-factor structure [29].

To improve readability, the main manuscript presents the summarized factor loading results, while the full rotated pattern matrix is provided in Supplementary Table S5. The six-factor solution was conceptually consistent with the framework developed through literature review, item mapping, and Delphi consultation. The Practice factor contained the largest number of items because it covered the core daily work of newly recruited nurses in MCH hospitals, including assessment, communication, care planning, intervention implementation, documentation, evaluation, continuity of care, and early risk recognition. In contrast, Health Advocacy and Equity contained fewer items because it was framed as a focused foundational domain involving awareness, communication, respect, and timely reporting rather than independent system-level advocacy. However, the close correspondence between the extracted factors and the pre-existing item blocks should be interpreted cautiously, as it may partly reflect conceptual preassignment, wording similarity, or questionnaire organization. The size and breadth of the Practice factor also suggest the need for further examination of possible subdomains and item reduction.

(2) Confirmatory factor analysis

**Figure 1:**
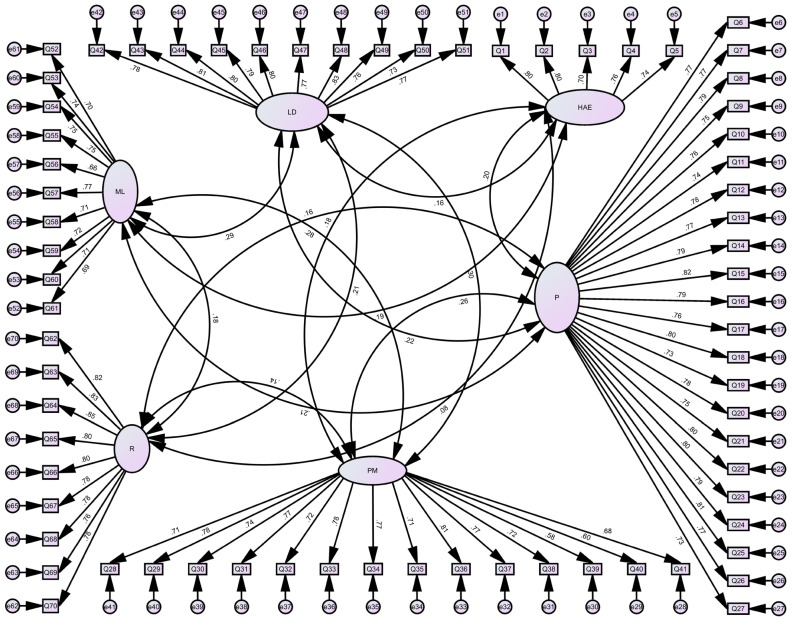
A schematic diagram of standardized model fitting of the scale (n = 226). Note: LD = Learning and Development; ML = Management and Leadership; R = Research; PM = Professional Morale; P = Practice; HAE = Health Advocacy and Equity. Single-headed arrows indicate standardized factor loadings, and double-headed arrows indicate correlations between latent variables. Light purple ovals represent latent variables, and black rectangles represent observed items.

The second subset of 226 samples was then subjected to CFA using maximum likelihood (ML) estimation to verify the factor structure derived from EFA [25]. As shown in Table 3, the first-order correlated six-factor model showed acceptable absolute fit indices, including χ^2^/df = 1.675, SRMR = 0.0595, and RMSEA = 0.055. However, the incremental fit indices were marginal, with CFI = 0.866 and TLI = 0.861. Therefore, the CFA results provide preliminary rather than definitive support for the proposed structure. The standardized path diagram of the first-order six-factor model is presented in Figure 1.

A second-order CFA model was also tested as a theoretically meaningful alternative, with the six first-order domains loading onto an overarching context-specific competency factor. Although this model showed acceptable χ^2^/df and RMSEA values, its incremental fit indices were lower than those of the first-order correlated six-factor model. Therefore, the first-order correlated six-factor model was retained as the preferred model. The relatively complex 70-item structure may have contributed to the strained incremental fit and indicates the need for future item reduction and cross-validation [31,34].

#### 3.2.4. Convergent and Discriminant Validity Analysis

Convergent and discriminant validity were evaluated using the CFA model with the 226-sample subset. To improve readability, the full item-level standardized factor loadings from CFA are provided in Supplementary Table S6. The main manuscript reports the loading range, AVE, and CR for each domain. The CR values ranged from 0.872 to 0.971, and the AVE values ranged from 0.519 to 0.642. Although the CR values suggested adequate internal consistency, some high CR values may partly reflect item overlap due to the large number of items within certain domains. In addition, several standardized factor loadings were comparatively lower, particularly in the Professional Morale and Management and Leadership domains. Therefore, the convergent validity evidence should be interpreted as preliminary rather than strong.

Discriminant validity was evaluated using the Fornell–Larcker criterion, which involved comparing the square root of the AVE value for each dimension with the corresponding correlations between dimensions. According to Fornell and Larcker [32], discriminant validity was achieved when the square root of the AVE of each dimension was greater than its correlations with other dimensions. The square root of each AVE value was greater than the corresponding inter-factor correlations, supporting discriminant validity according to the Fornell–Larcker criterion (Table 4). Discriminant validity results suggested that conceptually related domains, such as Professional Morale, Learning and Development, and Management and Leadership, were not statistically interchangeable. In practical terms, Professional Morale mainly reflected professional responsibility, ethical awareness, and role commitment; Learning and Development reflected self-directed learning, reflection, and continuing development; and Management and Leadership reflected work organization, team communication, quality improvement participation, and problem reporting.

#### 3.2.5. Reliability Analysis

The 70-item NCF-MCH showed high internal consistency, with a total Cronbach’s α of 0.944. Domain-level Cronbach’s α coefficients ranged from 0.875 to 0.968 (Table 5). However, the very high α coefficient for the Practice domain (0.968) should be interpreted cautiously, as values approaching 1 may indicate item redundancy, excessive content homogeneity, or overlapping item wording rather than superior psychometric quality alone. The total scale split-half reliability was 0.585, which was relatively low compared with the Cronbach’s α coefficients. This finding suggests that the total score may not function as a stable unidimensional indicator and should be interpreted cautiously. Domain-level scores may currently be more informative than the total score.

## 4. Discussion

This study developed a preliminary context-specific competency framework for newly recruited nurses in MCH hospitals and examined its initial psychometric properties using self-reported data. The final framework comprised six domains: Health Advocacy and Equity, Practice, Professional Morale, Learning and Development, Management and Leadership, and Research. These domains reflect competency expectations related to clinical care, family communication, professional conduct, early-career learning, teamwork, quality improvement, and evidence-informed practice.

The findings should be interpreted cautiously. The present study provides preliminary evidence of internal structure and reliability rather than definitive validation of the framework. The NCF-MCH has not yet been tested in terms of implementation feasibility, training effectiveness, clinical performance, workforce outcomes, or service capacity. Its current value lies mainly in offering an initial structure for describing self-perceived context-specific competency and identifying perceived training needs among newly recruited nurses in MCH hospitals.

### 4.1. Interpretation of the Structural Validity Findings

The exploratory factor analysis provided preliminary support for a six-factor structure. Principal axis factoring with Promax oblique rotation was used because the aim was to explore latent competency constructs and because competency domains were theoretically expected to be related. The six factors explained 59.01% of the variance, and the weak-to-modest factor correlations suggested that the domains were empirically distinguishable but conceptually related.

However, the EFA results should not be overinterpreted. Parallel analysis and minimum average partial criteria were not performed, and the six-factor solution closely corresponded to the pre-existing item blocks. This pattern may partly reflect conceptual preassignment, item wording similarity, or questionnaire organization [28]. In addition, the Practice domain contained 22 items and represented the most dominant part of the instrument. Although this reflects the centrality of clinical practice in the daily work of newly recruited nurses in MCH hospitals, it may also indicate that the current tool is weighted more heavily toward traditional clinical practice competencies than toward transversal or contextual dimensions, such as health advocacy, leadership, quality improvement, and research participation. Future studies should examine whether the Practice domain can be subdivided, shortened, or better balanced with other domains.

The CFA results also provided only partial support for the proposed structure. Although the first-order correlated six-factor model showed acceptable absolute fit indices, the CFI and TLI values remained below conventional criteria for good fit. Therefore, the proposed structure should be regarded as preliminary rather than definitively confirmed. The relatively complex 70-item model may have contributed to the strained incremental fit, but this complexity also reflects a limitation of the current instrument design [35]. A second-order CFA model was additionally tested, but it did not improve model fit. Thus, the NCF-MCH may be better interpreted as a multidimensional framework composed of related but distinguishable competency domains rather than as a strongly hierarchical structure dominated by one general competency factor.

### 4.2. Interpretation of Reliability and Item-Level Findings

The reliability evidence was mixed. Cronbach’s α coefficients suggested acceptable to high internal consistency for the overall scale and most domains, but the total scale split-half reliability was weak. Therefore, the total score should be interpreted cautiously, and domain-level scores may currently be more informative than the overall score.

The very high Cronbach’s α coefficient for the Practice domain may indicate redundancy or substantial content overlap among practice-related items [36]. This concern is consistent with the large number of items in this domain. In addition, several items showed corrected item-total correlations below 0.30, particularly in Health Advocacy and Equity, Management and Leadership, and Research. These items were retained because they represented theoretically and contextually important areas identified through literature review, source mapping, and Delphi consultation. Nevertheless, their relatively weak item-level performance suggests that further item reduction, short-form development, and modern psychometric analyses such as item response theory or Rasch analysis are needed.

### 4.3. Contextual and Developmental Interpretation of the Framework

The contribution of the NCF-MCH should be understood as a context-adapted and developmentally calibrated application of existing competency concepts rather than as a completely new competency theory. Compared with general nursing competency instruments [37], the NCF-MCH focuses on newly recruited nurses within two years of employment and translates broad competency concepts into MCH-specific task- and behaviour-level self-assessment items. It also reframes advanced areas such as leadership, research, and health advocacy as foundational or participatory behaviours rather than independent expert-level responsibilities.

The discriminant validity findings suggest that the NCF-MCH does not simply measure one undifferentiated competency construct. For example, Professional Morale, Learning and Development, and Management and Leadership are all related to early-career professional development, but they reflect different emphases: professional responsibility and role commitment, self-directed learning and reflection, and early participation in work organization, communication, problem reporting, and quality improvement. These differentiated domains may help describe different developmental functions during professional transition.

Benner’s novice-to-expert theory was used to define the expected proficiency boundary rather than to establish a full stage-based developmental scale [14]. Because the target population was newly recruited nurses within two years of employment, the items were positioned mainly at the novice-to-advanced beginner level, with some indicators reflecting transition toward early competent practice under supervision. This interpretation is particularly important for Management and Leadership and Research, which were framed as entry-level participatory behaviours such as reporting problems, assisting with team communication, participating in quality improvement, applying evidence under guidance, and supporting data collection. These domains should be further examined through cognitive interviews, frontline nurse feedback, supervisor ratings, and objective performance-based validation.

### 4.4. Practical Implications with Caution

The practical implications of this study should be considered preliminary. At this stage, the NCF-MCH may provide a structured self-assessment aid for describing self-perceived competency, clarifying role expectations during early employment, and identifying perceived training needs among newly recruited nurses in MCH hospital settings. Nurse managers and educators may use domain-level scores to identify areas in which newly recruited nurses perceive lower preparedness and to inform formative support, such as targeted mentoring, supervised practice opportunities, case-based learning, and individualized training plans.

However, no empirically validated competency thresholds, cut-off scores, or minimum passing standards were established in this study. Therefore, the NCF-MCH should not yet be used as a standalone tool for formal performance evaluation, certification, workforce policy decisions, or service improvement assessment. In addition, because the current version contains 70 items, routine use in busy clinical settings may be limited by administration time, respondent burden, and institutional feasibility. Future studies should examine administration time, user acceptability, feasibility in different institutional contexts, short-form development, and evidence-based scoring thresholds.

### 4.5. Limitations and Future Research

Several limitations should be acknowledged. First, this study used convenience sampling, and the validation sample was drawn from four MCH hospitals in Eastern China. Therefore, the findings may not be generalisable to other regions, hospital levels, institutional contexts, or healthcare systems. Although the framework was informed by international competency framework logic, including WHO competency frameworks and Mills’ terminology, the item content and expected role boundaries may still reflect the cultural, organizational, and professional characteristics of MCH nursing practice in China. International applicability should therefore not be assumed without cross-cultural adaptation, translation validation, and external testing in diverse healthcare contexts [38].

Second, the framework was validated using self-reported data. Newly recruited nurses may have limited experience in accurately judging their own competence, and responses may be influenced by social desirability bias or individual differences in interpreting competency levels. Therefore, the scores should be interpreted as self-perceived context-specific competency rather than directly observed clinical performance. No supervisor ratings, mentor evaluations, objective performance assessments, simulation scores, clinical outcomes, training outcomes, or criterion validity evidence were included in this stage.

Third, the psychometric evidence remains preliminary. The CFA sample size was limited relative to the complexity of the 70-item model, and the marginal incremental fit indices indicate that the proposed structure requires further confirmation. Criterion-related validity, predictive validity, responsiveness, measurement invariance, and comparison with existing competency scales were not examined. In addition, the EFA solution closely corresponded to the pre-existing item blocks, and the Practice factor contained 22 items, suggesting possible conceptual preassignment, item wording similarity, domain imbalance, and item redundancy.

Finally, limitations in the development process should also be noted. The Delphi panel was relatively management-oriented, which may have introduced conceptual bias toward managerial expectations rather than fully capturing frontline newly recruited nurses’ lived experiences, perceived difficulties, and transition challenges. Although newly recruited nurses provided feedback during the pilot test, they were not included as formal Delphi voting members. The pilot test was also conducted with a small sample from a single hospital, and the revised version was not subjected to a second independent pilot test. Future studies should include more diverse stakeholder groups and larger independent samples, apply multi-source and objective validation methods, examine measurement invariance and responsiveness, and conduct item reduction or short-form development to improve parsimony and feasibility.

## 5. Conclusions

This study developed the NCF-MCH and preliminarily validated its corresponding self-assessment tool. By translating general nursing competency concepts into MCH-specific, task-linked, and developmentally appropriate self-assessment items, the study provides an initial methodological example of how a contextualized competency framework can be adapted for a specific nursing population and service setting. The NCF-MCH helps clarify competency expectations for newly recruited nurses in MCH hospitals and may serve as an initial reference for identifying perceived training needs, informing onboarding programs, and supporting competency-based education in MCH nursing. The contextualized nature of the NCF-MCH is both its strength and a boundary of its generalizability. Because the item content and expected role boundaries may reflect the cultural, organizational, and professional characteristics of MCH nursing practice in China, the framework should not be directly generalized to other healthcare systems without cross-cultural adaptation and external validation. Further external, longitudinal, cross-cultural, and objective validation is required before the tool can be used for formal performance evaluation, workforce policy decisions, or service improvement assessment.

## Figures and Tables

**Table 1:** Characteristics of the Delphi Panel (n = 23).

Item	Category	n	Percentage (%)
Gender	Male	3	13
	Female	20	87
Age (years)	30–39	7	30.4
	40–49	15	65.2
	≥50	1	4.3
Education level	Master’s degree	8	34.8
	Bachelor’s degree	15	65.2
Years of work experience	10–19 years	16	69.6
	20–29 years	6	26.1
	30–40 years	1	4.3
Professional title	Senior	2	8.7
	Associate senior	15	65.2
	Intermediate	6	26.1
Administrative position	Director of Nursing Department	1	4.3
	Deputy Director of Nursing Department	1	4.3
	Head nurse	17	73.9
	Teaching secretary	4	17.4
Research field (Multiple responses allowed)	Nursing management	10	43.5
	Nursing education	6	26.1

**Table 2:** Socio-demographic characteristics of participants (n = 554).

Characteristics	Categories	n	%
Gender	Male	38	6.9
	Female	516	93.1
Age (years)	21~24	387	69.9
	25~28	167	30.1
Length of nursing experience (months)	0~6	180	32.5
	7~12	136	24.5
	13~18	105	19.0
	19~24	133	24.0
Educational Background	Junior College	201	36.3
	Undergraduate	326	58.8
	Graduate	27	4.9
Obstetrics and Pediatrics Rotation during Internship	yes	410	74
	no	144	26

**Table 3:** Fit indices of confirmatory factor analysis (n = 226).

Models	χ^2^/df	SRMR	RMSEA	TLI	CFI
One-factor model	4.398	0.1905	0.123	0.298	0.318
First-order six-factor model	1.675	0.0595	0.055	0.861	0.866
Second-order six-factor model	1.795	0.0778	0.059	0.836	0.841

Note: SRMR: standardized root mean square residual; RMSEA: Root Mean Square Error of Approximation; TLI: Tucker–Lewis Index; CFI: Comparative Fit Index.

**Table 4:** Discriminant validity of the scale (n = 226).

	P	PM	ML	LD	R	HAE
P	0.775 ^a^					
PM	0.260	0.726 ^a^				
ML	0.292	0.288	0.720 ^a^			
LD	0.260	0.285	0.292	0.784 ^a^		
R	0.292	0.281	0.288	0.292	0.801 ^a^	
HAE	0.292	0.281	0.281	0.281	0.281	0.760 ^a^

^a^ Represents the square root of the average variance extracted values of each dimension.

**Table 5:** Reliability coefficients of the overall scale and each dimension.

Dimension	No. of Items	Cronbach’s α	Split-Half Reliability	Test–Retest
Health Advocacy and Equity	5	0.875	0.855	0.744
Practice	22	0.968	0.956	0.871
Professional Morale	14	0.940	0.922	0.832
Learning and Development	10	0.935	0.925	0.986
Management and Leadership	10	0.936	0.944	0.991
Research	9	0.936	0.917	0.982
Total scale	70	0.944	0.585	0.956

## Data Availability

The datasets used and/or analyzed during the current study are available from the corresponding author on reasonable request, due to privacy and institutional restrictions.

## Supplementary Materials

The following supporting information can be downloaded at https://www.mdpi.com/article/10.3390/healthcare14121772/s1, Supplementary Table S1. Included Literature and Framework Documents for Framework Development and Item Generation. Supplementary Table S2. Item–Source Mapping and Operationalisation Process for the Final 70 Tertiary Indicators. Supplementary Table S3. Major Item Revisions After Pilot Testing and Frontline User Review. Supplementary Table S4. Item-Level Analysis of the Final 70-Item NCF-MCH. Supplementary Table S5. Rotated Pattern Matrix from Exploratory Factor Analysis. Supplementary Table S6. Standardized Factor Loadings from Confirmatory Factor Analysis.

## Abbreviations

The following abbreviations are used in this manuscript:

MCH	Maternal and Child Health
NCF-MCH	Newly recruited nurses’ Competency framework in maternal and child health hospitals
WHO	World Health Organization
CVI	Content Validity Index
I-CVI	Item-level CVI
S-CVI	Scale-level CVI
KMO	Kaiser–Meyer–Olkin
EFA	Exploratory Factor Analysis
CFA	Confirmatory Factor Analysis
SRMR	Standardized Root Mean Square Residual
RMSEA	Root Mean Square Error of Approximation
CFI	Comparative Fit Index
TLI	Tucker–Lewis Index
ICC	Intraclass correlation coefficient
CR	Composite Reliability
AVE	Average Variance Extracted
