# Supplementary material for: Construction and Validation of a Contextualized Competency Framework for Newly Recruited Nurses in Maternal and Child Health Hospitals
Source: Healthcare (Basel). 2026 Jun 19;14(12):1772. doi: 10.3390/healthcare14121772 (PMC13299856; doi:10.3390/healthcare14121772)
Supplement: Supplementary file 1 [file healthcare-14-01772-s001.zip › Supplementary_Table S1 Included Literature and Framework Documents for Framework Development and Item Generation.pdf]

Supplementary Table S1. Included Literature and Framework Documents for Framework Development and Item Generation

Note. The 23 sources were classified according to their primary role in framework development. Some documents provided direct MCH-related item content, whereas others provided general nursing competency content or conceptual/structural guidance for organizing domains, competencies, behaviours, practice activities, and tasks. The main contributions are summarized based on available source documents, abstracts, and extracted indicator tables; Supplementary Table S2 presents the primary source basis and contextual operationalisation of each final item.

No.	Source	Year / Type	Target population or context	Role in framework development	Main contribution to item generation
1	World Health Organization. Defining competent maternal and newborn health professionals. Geneva: WHO.	2018 / WHO technical document	Maternal and newborn health professionals	Direct item-content source	Provided competency expectations for skilled maternal and newborn health professionals, supporting maternal-newborn care, professional responsibilities, teamwork, communication, quality, and safe practice content.
2	International Confederation of Midwives. Essential Competencies for Midwifery Practice. The Hague: ICM.	2024 / International competency framework	Midwifery practice and maternal-newborn care	Adjacent MNH professional competency source	Provided adjacent maternal-newborn professional competency content, including antenatal, intrapartum, postnatal, newborn, reproductive health, professional responsibility, communication, and evidence-informed practice.
3	International Council of Nurses. ICNP Catalogue: Prenatal Nursing Care. Geneva: ICN.	2020 / Nursing terminology catalogue	Prenatal nursing care	Direct item-content source	Provided standardized prenatal nursing terminology, including prenatal nursing diagnoses, interventions, outcomes, and care activities relevant to MCH self-assessment items.
4	National Certification Corporation. Maternal Newborn Nursing Content Outline. Chicago: NCC.	2025 / Certification content outline	Maternal-newborn nursing	Direct item-content source	Provided maternal-newborn nursing certification content areas, including maternal assessment, postpartum care, newborn care, complications, patient education, and professional role expectations.
5	NHS England. Maternity and Neonatal Core Competency Framework (v2). London: NHS England.	2023 / National competency framework	Maternity and neonatal services	Direct item-content source	Provided maternity and neonatal core competency areas, including safety, communication, escalation, safeguarding, multidisciplinary working, professional practice, and role-specific maternity/neonatal capabilities.
6	Council of International Neonatal Nurses. Neonatal Nursing Core Competencies.	2021 / International neonatal nursing competency framework	Neonatal nursing; novice-to-expert neonatal nurse development; newborns and families	Direct item-content source	Provided neonatal nursing competency content for novice to expert practice, including newborn assessment, neonatal safety, family-centered developmental care, breastfeeding and feeding support, escalation/referral, mentorship, leadership, research, and professional development.
7	Nurse-Family Partnership International. International NFP Core Competencies. Denver: NFP International.	2021 / Program competency framework	Maternal-child home visiting and family support	Adjacent MCH contextual source	Provided family-centered and home-visiting competency content, including health promotion, family education, relational communication, follow-up support, maternal-child development, and strengths-based practice.
8	World Health Organization. Global Competency and Outcomes Framework for Universal Health Coverage. Geneva: WHO.	2022 / WHO competency framework	Health workforce for universal health coverage	Conceptual / structural framework source	Provided a structural reference for organizing competency requirements into domains, competencies, behaviours, practice activities, tasks, and outcomes within a UHC-oriented framework.

No.	Source	Year / Type	Target population or context	Role in framework development	Main contribution to item generation
9	World Health Organization. Rehabilitation Competency Framework. Geneva: WHO.	2021 / WHO competency framework	Rehabilitation workforce	Conceptual / structural framework source	Provided a structural example of contextualized competency framework development and the translation of broad competency domains into activities, behaviours, and task-linked indicators.
10	World Health Organization. Eye Care Competency Framework. Geneva: WHO.	2022 / WHO competency framework	Eye care workforce	Conceptual / structural framework source	Provided a structural example of adapting competency domains, behaviours, practice activities, and tasks to a specialized service context.
11	Lu J, Deng B, Liu J. Construction of a pediatric nurse competency model based on exploratory factor analysis.	2016 / Chinese-language empirical study	Pediatric nurses	Direct item-content source	Provided pediatric nurse competency dimensions and indicators, including risk management, care and concern, professional ethics, communication and coordination, coping capacity, social learning, and problem-solving ability.
12	Lu J, Liu J, Deng B. Construction of an obstetric and gynecological nurse competency model based on exploratory factor analysis.	2017 / Chinese-language empirical study	Obstetric and gynecological nurses	Direct item-content source	Provided obstetric and gynecological nurse competency dimensions and items, including professional quality, psychological quality, professional attitude, interpersonal communication, stress tolerance, ethical quality, teamwork, and emergency response ability.
13	Chen M. Construction and empirical study of a core competency system for T-shaped nursing talents in tertiary Grade A hospitals under the Healthy China strategy.	2024 / Chinese-language dissertation	General tertiary hospital nursing workforce	General nursing competency source	Provided broader tertiary-hospital nursing competency domains, including clinical nursing, leadership and management, humanistic practice, nursing research, clinical thinking, professional development, professional values, and personal attributes.
14	Zhang J, Ye W, Fan F. Development of a self-assessment tool for measuring competences of obstetric nurses in rooming-in wards in China.	2015 / Scale development study	Obstetric nurses in rooming-in wards	Direct item-content source	Provided self-assessment content and competency domains for obstetric nurses in rooming-in wards, supporting maternal-infant care, communication, professional responsibility, and ward-based nursing practice items.
15	Huang Y, Wang Z, Zhang D, et al. Construction and application of a nursing recruitment indicator system for maternal and child health hospitals based on a competency model.	2025 / Chinese-language applied study	Nursing recruitment in MCH hospitals	Direct item-content source	Provided MCH hospital nursing recruitment indicators covering basic nursing ability, health management, evidence-based practice, communication, professional culture, ethics, condition assessment, and health education.
16	Zhang J. Study on a competency evaluation model for responsible nurses in obstetric rooming-in wards.	2013 / Chinese-language dissertation	Responsible nurses in obstetric rooming-in wards	Direct item-content source	Provided competency indicators for responsible nurses in obstetric rooming-in wards, including maternal-infant knowledge, clinical skills, risk intervention, coordination, emergency response, health education, interpersonal communication, professional quality, and self-development.
17	Paterson K E, Leff E W, Luce M M, et al. From the field: a maternal-child health nursing competence validation model.	2004 / MCH nursing competency validation study	Maternal-child health nursing	Direct item-content source	Provided an MCH nursing competence validation model and competency areas relevant to maternal-child health nursing roles, professional development, and practice-based competency evaluation.
18	Ren L, Zhao X. Construction of a competency model and indicator system for new nurses in specialized maternal and child health hospitals based on the Onion Model.	2021 / Chinese-language framework study	New nurses in specialized MCH hospitals	Direct item-content source	Provided a competency model for new nurses in specialized MCH hospitals, covering knowledge, skills, ability, and professional literacy, including MCH specialist knowledge, emergency care, infection control, technical skills, clinical thinking, communication, and career development.

No.	Source	Year / Type	Target population or context	Role in framework development	Main contribution to item generation
19	Yang Y, Li H, Li X, et al. Construction of a competency evaluation indicator system for 'nursing + maternal-infant care' nurses.	2024 / Chinese-language indicator-system study	Maternal-infant care nurses	Direct item-content source	Provided competency indicators for maternal-infant care nurses, including maternal and infant knowledge, pregnancy and postpartum care, newborn and infant care, postpartum recovery, critical thinking, education and consultation, coordination, professional development, ethics, and service awareness.
20	Zheng G, Guo Y, Zhang L, et al. Construction of a training system for maternal-infant nursing specialist nurses in Henan Province.	2023 / Chinese-language training-system study	Maternal-infant nursing specialist nurses	Adjacent MCH specialist training source	Provided training-system content for maternal-infant nursing specialist nurses, including theoretical knowledge, maternal and newborn care skills, delivery and postpartum care, infant care, nursing quality and safety, documentation, and management/coordination abilities.
21	Chen Q, Kong L, Jiang S, et al. Construction of entrusted professional activity indicators for newly recruited nurses.	2023 / Chinese-language indicator-system study	Newly recruited nurses	General newly recruited nurse competency source	Provided entrusted professional activity indicators for newly recruited nurses, including admission and transfer assessment, nursing diagnosis and prioritization, care planning, nursing interventions and evaluation, common procedures, emergency response, reporting and documentation, psychological support, handover, and health education.
22	Englander R, Cameron T, Ballard A J, et al. Toward a common taxonomy of competency domains for the health professions and competencies for physicians.	2013 / Health professions taxonomy paper	Health professions education	Conceptual / structural framework source	Provided a common competency-domain taxonomy for health professions, supporting the conceptual organization of professional competence across broad domains and competency statements.
23	Liu M, Kunaiktikul W, Senaratana W, et al. Development of competency inventory for registered nurses in the People's Republic of China: scale development.	2007 / Scale development study	Registered nurses in China	General nursing competency source	Provided a Chinese registered-nurse competency inventory and scale-development reference, supporting broader nursing competency domains such as clinical care, communication, professionalism, critical thinking, leadership, teaching, and research-related competence.

Abbreviations: COINN = Council of International Neonatal Nurses; MCH = maternal and child health; WHO = World Health Organization; ICN = International Council of Nurses; ICM = International Confederation of Midwives; NCC = National Certification Corporation; NHS = National Health Service; NFP = Nurse-Family Partnership; UHC = Universal Health Coverage.
