# Supplementary material for: Construction and Validation of a Contextualized Competency Framework for Newly Recruited Nurses in Maternal and Child Health Hospitals
Source: Healthcare (Basel). 2026 Jun 19;14(12):1772. doi: 10.3390/healthcare14121772 (PMC13299856; doi:10.3390/healthcare14121772)
Supplement: Supplementary file 1 [file healthcare-14-01772-s001.zip › Supplementary_Table_S2_Item_Source_Mapping_Operationalisation_with_ICVI.pdf]

Supplementary Table S2. Item–Source Mapping and Operationalisation Process for the Final 70 Tertiary Indicators

Purpose. This table documents how source concepts were transformed into MCH-related, observable, and self-assessable item wording for the final 70 tertiary indicators retained after Delphi consultation. The table is intended to demonstrate the theoretical, empirical, and contextual basis of the finalized items, rather than to document every interim item generated before Delphi consultation.

Operationalisation logic. Mills' reconceptualised competency terminology was used as an organizing and operational logic rather than as a rigid label assigned to every final item. Broad competency concepts were linked to MCH-related practice activities and then rewritten as observable behaviour- or task-level statements. Benner's novice-to-expert theory was used to calibrate the expected proficiency boundary for newly recruited nurses within two years of employment.

Mapping element	Description
Source basis	General nursing, health workforce, MCH, maternal-newborn, neonatal, prenatal, child health, and related competency documents or studies.
Extracted competency concept	The broad concept, competency, capability, practice activity, or task identified from the source material.
MCH-related practice activity	The maternal, neonatal, pediatric, women's health, family education, breastfeeding support, risk identification, interprofessional collaboration, or continuity-of-care context in which the concept is enacted.
Operationalized behaviour/task	The final observable and self-assessable item wording used in the corresponding self-assessment tool.
Benner-informed proficiency expectation	The expected level was framed primarily at the novice-to-advanced beginner stage, with some transition toward early competent practice under supervision.
Adaptation decision	Whether the item was retained, contextualized, reframed, merged, or added after literature review, research-team review, Delphi consultation, and pilot feedback.
**Item-level content validity index (I-CVI)**	Proportion of experts rating each final item as relevant (3 or 4 on a 4-point scale); S-CVI/Ave = 0.94.

Item / Domain	Source basis	Extracted competency concept	MCH-related practice activity	Operationalized behaviour/task (final item wording)	Benner-informed proficiency expectation	Adaptation decision	I-CVI
1 / Health Advocacy and Equity	UHC C2.1; UHC C4.3; ECCF CAC3/CAA2; CIRN 54, 57; COINN; SBA/MNH; ICNP Prenatal; NCC MNN; NHS CCF	Enhancing community health awareness	Community MCH health education, health promotion, equity-oriented resource linkage for women, children, and families.	Deliver professional health knowledge (e.g., prenatal care, first aid for children, disease prevention) in plain, understandable, and interactive ways according to the needs of different maternal and child health groups, such as pregnant and postpartum women, children, and women.	Foundational participation in health education and resource linkage; activities may be performed with support rather than independently led.	Adapted for MCH community and equity context	0.93
2 / Health Advocacy and Equity	UHC C3.4-C3.5; UHC C9.3/C10.1; RCF P-C3; CIRN 11, 32, 34; COINN; SBA/MNH; ICNP Prenatal; NCC MNN; NHS CCF	Cross-cultural communication	Community MCH health education, health promotion, equity-oriented resource linkage for women, children, and families.	Communicate effectively with clients from different backgrounds (age, culture, occupation, and region), such as older adults, young mothers, and rural women; respect cultural and belief differences; and address conflicts in health understanding.	Foundational participation in health education and resource linkage; activities may be performed with support rather than independently led.	Adapted for MCH community and equity context	0.93
3 / Health Advocacy and Equity	UHC C3.6/C4.5; ECCF CAC2/CAC4; CIRN 30, 45, 48; COINN; SBA/MNH; ICNP Prenatal; NCC MNN; NHS CCF	Integrating equity into MCH service practice	Community MCH health education, health promotion, equity-oriented resource linkage for women, children, and families.	Attend to vulnerable maternal and child health groups, such as rural women, migrant populations, and families of children with disabilities; connect them with health resources, such as free screening and charitable assistance; and promote equitable access to services.	Foundational participation in health education and resource linkage; activities may be performed with support rather than independently led.	Adapted for MCH community and equity context	0.93
4 / Health Advocacy and Equity	UHC C4.3; ECCF CAC3/CAA2; CIRN 50, 54, 57; COINN; SBA/MNH; ICNP Prenatal; NCC MNN; NHS CCF	MCH health promotion and grassroots service practice	Community MCH health education, health promotion, equity-oriented resource linkage for women, children, and families.	Actively participate in community maternal and child health promotion activities organized by the hospital, such as reproductive health, sexually transmitted diseases, breastfeeding, and child nutrition management.	Foundational participation in health education and resource linkage; activities may be performed with support rather than independently led.	Adapted for MCH community and equity context	0.93
5 / Health Advocacy and Equity	UHC C2.1/C4.3/C4.4; ECCF CAC2/CAA2; CIRN 45, 54, 57; COINN; SBA/MNH; ICNP Prenatal; NCC MNN; NHS CCF	MCH health promotion and grassroots service practice	Community MCH health education, health promotion, equity-oriented resource linkage for women, children, and families.	Actively participate in public welfare activities and community, campus, or enterprise collaborations, and proactively provide grassroots health services to improve public health literacy.	Foundational participation in health education and resource linkage; activities may be performed with support rather than independently led.	Adapted for MCH community and equity context	0.93
6 / Nursing Practice	RCF P-C1; ECCF PC1.1; UHC C1; CIRN 16, 19, 43; COINN; SBA/MNH; ICNP Prenatal; NCC MNN; NHS CCF	Being patient- and family-centered in practice	Woman-, child-, and family-centred communication and ethical clinical interaction in MCH care settings.	Think from the perspective of patients and their family members.	Demonstrates basic interpersonal, ethical, and communication behaviours appropriate to early-career practice.	Retained and contextualized	1.00
7 / Nursing Practice	UHC C2.2/C5.1; ECCF PC1.2/PA5; CIRN 16, 43,	Being patient- and family-centered in	Woman-, child-, and family-centred communication and	Provide sufficient information to help patients and their family members make decisions.	Demonstrates basic interpersonal, ethical, and	Retained and contextualized	1.00

Item / Domain	Source basis	Extracted competency concept	MCH-related practice activity	Operationalized behaviour/task (final item wording)	Benner-informed proficiency expectation	Adaptation decision	I-CVI
	54; COINN; SBA/MNH; ICNP Prenatal; NCC MNN; NHS CCF	practice	ethical clinical interaction in MCH care settings.		communication behaviours appropriate to early-career practice.		
8 / Nursing Practice	RCF P-C5.1; UHC C21; CIRN 47; COINN; SBA/MNH; NCC MNN; NHS CCF	Working within the scope of practice and competence	Woman-, child-, and family-centred communication and ethical clinical interaction in MCH care settings.	Maintain awareness of one's own limitations when providing care for patients and their families.	Recognizes own limits and seeks senior support when situations exceed experience or competence.	Reframed for newly recruited nurse role	1.00
9 / Nursing Practice	RCF P-C5.2; UHC C16.2/C21; CIRN 29, 30, 47; COINN; SBA/MNH; NCC MNN; NHS CCF	Working within the scope of practice and competence	Woman-, child-, and family-centred communication and ethical clinical interaction in MCH care settings.	Seek support and guidance when encountering situations beyond one's scope of practice or competence.	Recognizes own limits and seeks senior support when situations exceed experience or competence.	Reframed for newly recruited nurse role	1.00
10 / Nursing Practice	RCF P-C4.1; UHC C5.2-C5.5; CIRN 4, 5, 13; COINN; SBA/MNH; NCC MNN; NHS CCF	Solving problems and making decisions with a scientific and rigorous attitude	Woman-, child-, and family-centred communication and ethical clinical interaction in MCH care settings.	Integrate information from multiple sources and consider complex personal, environmental, and health-related factors before making decisions.	Demonstrates basic interpersonal, ethical, and communication behaviours appropriate to early-career practice.	Retained and contextualized	1.00
11 / Nursing Practice	RCF P-C4.3; UHC C7.3; CIRN 2; COINN; SBA/MNH; NCC MNN; NHS CCF	Solving problems and making decisions with a scientific and rigorous attitude	Woman-, child-, and family-centred communication and ethical clinical interaction in MCH care settings.	Identify innovative solutions when addressing challenges together with patients.	Demonstrates basic interpersonal, ethical, and communication behaviours appropriate to early-career practice.	Retained and contextualized	1.00
12 / Nursing Practice	RCF P-C2.1; UHC C15.1-C15.2; ECCF PC1.3; CIRN 19, 36; COINN; SBA/MNH; ICNP Prenatal; NCC MNN; NHS CCF	Establishing collaborative relationships with patients and families	Woman-, child-, and family-centred communication and ethical clinical interaction in MCH care settings.	Establish good and mutually trusting nurse-patient relationships with service recipients.	Demonstrates basic interpersonal, ethical, and communication behaviours appropriate to early-career practice.	Retained and contextualized	1.00
13 / Nursing Practice	RCF P-C2.3; UHC C15.3; CIRN 38, 40, 43; COINN; SBA/MNH; NCC MNN; NHS CCF	Establishing collaborative relationships with patients and families	Woman-, child-, and family-centred communication and ethical clinical interaction in MCH care settings.	Maintain ethical boundaries with service recipients and relevant personnel in clinical practice.	Demonstrates basic interpersonal, ethical, and communication behaviours appropriate to early-career practice.	Retained and contextualized	1.00
14 / Nursing Practice	RCF P-C2.2; UHC C15.4; CIRN 43, 45; COINN; SBA/MNH; NCC MNN; NHS CCF	Establishing collaborative relationships with patients and families	Woman-, child-, and family-centred communication and ethical clinical interaction in MCH care settings.	Recognize and minimize power imbalances between patients and nursing staff.	Demonstrates basic interpersonal, ethical, and communication behaviours appropriate to early-career practice.	Retained and contextualized	1.00
15 / Nursing Practice	RCF P-C3.1/P-C3.2; UHC C9.3/C10.1; CIRN 11, 32, 34; COINN; SBA/MNH; ICNP Prenatal; NCC MNN; NHS CCF	Maintaining effective communication with patients and families	Woman-, child-, and family-centred communication and ethical clinical interaction in MCH care settings.	Understand the different communication needs of service recipients based on age, gender, culture, and other backgrounds; adjust communication methods to ensure smooth information delivery and genuine understanding.	Demonstrates basic interpersonal, ethical, and communication behaviours appropriate to early-career practice.	Retained and contextualized	1.00
16 / Nursing Practice	RCF P-C3.2/P-C3.3; UHC C10.4/C10.5; ECCF PC5.2; CIRN 32, 54, 57; COINN; SBA/MNH; ICNP Prenatal; NCC MNN; NHS CCF	Maintaining effective communication with patients and families	Woman-, child-, and family-centred communication and ethical clinical interaction in MCH care settings.	Use language that service recipients can understand whenever possible, and use translation or modern technological devices when necessary.	Demonstrates basic interpersonal, ethical, and communication behaviours appropriate to early-career practice.	Retained and contextualized	1.00
17 / Nursing Practice	RCF P-C3.5; UHC C9.5; CIRN 39, 44; COINN; SBA/MNH; ICNP Prenatal; NCC MNN; NHS CCF	Maintaining effective communication with patients and families	Woman-, child-, and family-centred communication and ethical clinical interaction in MCH care settings.	Consider environmental factors such as noise and privacy during communication.	Demonstrates basic interpersonal, ethical, and communication behaviours appropriate to early-career practice.	Retained and contextualized	1.00
18 / Nursing Practice	RCF P-A3.1; ECCF PA3.1; CIRN 5, 7, 13; COINN; SBA/MNH; ICNP Prenatal; NCC MNN; NHS CCF	Taking a health history	MCH assessment, care planning, intervention, documentation, discharge planning, and follow-up for maternal, neonatal, pediatric, and women's health services.	Fully understand the scope and complexity of determinants of individual health status, and obtain comprehensive health, environmental, and personal history information accordingly when taking a health history.	Performs routine assessment and care tasks within role boundaries; assists in complex planning or continuity tasks.	Retained and contextualized	1.00
19 / Nursing Practice	RCF P-A3.3; ECCF PA3; CIRN 7, 13; COINN; SBA/MNH; ICNP Prenatal; NCC MNN; NHS CCF	Conducting nursing assessment	MCH assessment, care planning, intervention, documentation, discharge planning, and follow-up for maternal, neonatal, pediatric, and women's health services.	Independently complete routine physical examinations.	Performs routine assessment and care tasks within role boundaries; assists in complex planning or continuity tasks.	Retained and contextualized	1.00
20 / Nursing Practice	RCF P-A3.2; CIRN 1, 12; COINN; SBA/MNH; ICNP Prenatal; NCC MNN; NHS CCF	Conducting nursing assessment	MCH assessment, care planning, intervention, documentation, discharge planning, and follow-up for	Assess whether service recipients are at risk of harming themselves and/or others, and implement protective strategies when appropriate.	Participates in assessment, protection, diagnosis, planning, and documentation under mentor/senior guidance	Reframed for newly recruited nurse role	1.00

Item / Domain	Source basis	Extracted competency concept	MCH-related practice activity	Operationalized behaviour/task (final item wording)	Benner-informed proficiency expectation	Adaptation decision	I-CVI
			maternal, neonatal, pediatric, and women's health services.		where needed.		
21 / Nursing Practice	RCF P-A4; ECCF PA7.1; CIRN 14, 16; COINN; SBA/MNH; ICNP Prenatal; NCC MNN; NHS CCF	Developing a nursing care plan	MCH assessment, care planning, intervention, documentation, discharge planning, and follow-up for maternal, neonatal, pediatric, and women's health services.	Assist in developing appropriate nursing interventions that can meet the goals of patients and their family members, including the expected timeframe.	Performs routine assessment and care tasks within role boundaries; assists in complex planning or continuity tasks.	Retained and contextualized	1.00
22 / Nursing Practice	RCF P-A6; ECCF PA8; CIRN 15; COINN; SBA/MNH; ICNP Prenatal; NCC MNN; NHS CCF	Implementing nursing interventions	MCH assessment, care planning, intervention, documentation, discharge planning, and follow-up for maternal, neonatal, pediatric, and women's health services.	Implement nursing interventions according to the nursing care plan.	Performs routine assessment and care tasks within role boundaries; assists in complex planning or continuity tasks.	Retained and contextualized	1.00
23 / Nursing Practice	UHC C2.1/C4.4; ECCF PA5; RCF P-A6; CIRN 19, 54, 57; COINN; SBA/MNH; ICNP Prenatal; NCC MNN; NHS CCF	Implementing nursing interventions	MCH assessment, care planning, intervention, documentation, discharge planning, and follow-up for maternal, neonatal, pediatric, and women's health services.	Provide routine health education and training for patients and their families to enhance their confidence in self-care and their level of self-management.	Performs routine assessment and care tasks within role boundaries; assists in complex planning or continuity tasks.	Retained and contextualized	1.00
24 / Nursing Practice	RCF P-A2/P-A3/P-A4; ECCF PA2/PA4/PA7; CIRN 5, 7, 14; COINN; SBA/MNH; ICNP Prenatal; NCC MNN; NHS CCF	Writing nursing records	MCH assessment, care planning, intervention, documentation, discharge planning, and follow-up for maternal, neonatal, pediatric, and women's health services.	Comprehensively analyze case information, including health history, physical examination, nursing assessment, and auxiliary examinations; make nursing diagnoses; summarize case characteristics; propose nursing plans; and complete nursing records.	Participates in assessment, protection, diagnosis, planning, and documentation under mentor/senior guidance where needed.	Reframed for newly recruited nurse role	1.00
25 / Nursing Practice	RCF P-A7; ECCF PA9; CIRN 14, 15, 18; COINN; SBA/MNH; ICNP Prenatal; NCC MNN; NHS CCF	Evaluating nursing progress	MCH assessment, care planning, intervention, documentation, discharge planning, and follow-up for maternal, neonatal, pediatric, and women's health services.	Determine whether the expected nursing goals have been achieved based on assessment results.	Performs routine assessment and care tasks within role boundaries; assists in complex planning or continuity tasks.	Retained and contextualized	1.00
26 / Nursing Practice	RCF P-A8; ECCF PA9; CIRN 16, 19, 30; COINN; SBA/MNH; ICNP Prenatal; NCC MNN; NHS CCF	Ensuring continuity of healthcare services	MCH assessment, care planning, intervention, documentation, discharge planning, and follow-up for maternal, neonatal, pediatric, and women's health services.	Develop discharge plans together with patients and their families, and provide support as needed.	Performs routine assessment and care tasks within role boundaries; assists in complex planning or continuity tasks.	Retained and contextualized	1.00
27 / Nursing Practice	RCF P-A8; ECCF PA9; CIRN 18, 30; COINN; SBA/MNH; ICNP Prenatal; NCC MNN; NHS CCF	Ensuring continuity of healthcare services	MCH assessment, care planning, intervention, documentation, discharge planning, and follow-up for maternal, neonatal, pediatric, and women's health services.	Conduct follow-up after discharge and provide further nursing services when necessary.	Performs routine assessment and care tasks within role boundaries; assists in complex planning or continuity tasks.	Retained and contextualized	1.00
28 / Professional Morale	RCF PM-C2; UHC C12.4/C8.3; CIRN 36, 42, 49; COINN; SBA/MNH; NCC MNN; NHS CCF	Maintaining professionalism	Professional, ethical, legal, collaborative, and respectful practice in MCH nursing.	Demonstrate confidence to service recipients at work.	Demonstrates routine professionalism, empathy, boundaries, collaboration, and self-regulation expected of a novice/advanced beginner.	Retained and contextualized	1.00
29 / Professional Morale	RCF core values; UHC C3.1; ECCF PMC1; CIRN 11, 19; COINN; SBA/MNH; ICNP Prenatal; NCC MNN; NHS CCF	Maintaining professionalism	Professional, ethical, legal, collaborative, and respectful practice in MCH nursing.	Show empathy toward patients and their families.	Demonstrates routine professionalism, empathy, boundaries, collaboration, and self-regulation expected of a novice/advanced beginner.	Retained and contextualized	1.00
30 / Professional Morale	RCF PM-C2/P-C2.3; UHC C15.3; CIRN 36, 38, 40; COINN; SBA/MNH; NCC MNN; NHS CCF	Maintaining professionalism	Professional, ethical, legal, collaborative, and respectful practice in MCH nursing.	Maintain clear professional boundaries with colleagues and stakeholders.	Demonstrates routine professionalism, empathy, boundaries, collaboration, and self-regulation expected of a novice/advanced beginner.	Retained and contextualized	1.00
31 / Professional Morale	RCF PM-C1; UHC C22; ECCF PMC2; CIRN 38, 40, 42; COINN; SBA/MNH;	Practicing within legal and regulatory frameworks	Professional, ethical, legal, collaborative, and respectful practice in MCH nursing.	Strictly comply with professional ethics, laws and regulations, rules, and institutional policies in daily work.	Demonstrates routine professionalism, empathy, boundaries, collaboration, and	Retained and contextualized	1.00

Item / Domain	Source basis	Extracted competency concept	MCH-related practice activity	Operationalized behaviour/task (final item wording)	Benner-informed proficiency expectation	Adaptation decision	I-CVI
	ICNP Prenatal; NCC MNN; NHS CCF				self-regulation expected of a novice/advanced beginner.		
32 / Professional Morale	UHC C13.4/C22; ECCF PA2/PMC2; CIRN 39, 44; COINN; SBA/MNH; ICNP Prenatal; NCC MNN; NHS CCF	Practicing within legal and regulatory frameworks	Professional, ethical, legal, collaborative, and respectful practice in MCH nursing.	Respect patient privacy.	Demonstrates routine professionalism, empathy, boundaries, collaboration, and self-regulation expected of a novice/advanced beginner.	Retained and contextualized	1.00
33 / Professional Morale	UHC C3.3/C10.3; ECCF PMC1/PMC4; CIRN 34, 38, 45; COINN; SBA/MNH; ICNP Prenatal; NCC MNN; NHS CCF	Practicing within legal and regulatory frameworks	Professional, ethical, legal, collaborative, and respectful practice in MCH nursing.	Recognize and strive to eliminate the potentially harmful effects of personal biases and beliefs, and identify and address existing or potential conflicts of interest.	Demonstrates routine professionalism, empathy, boundaries, collaboration, and self-regulation expected of a novice/advanced beginner.	Retained and contextualized	1.00
34 / Professional Morale	RCF PM-C3; UHC C16.1; CIRN 20, 24, 29; COINN; SBA/MNH; NCC MNN; NHS CCF	Working collaboratively	Professional, ethical, legal, collaborative, and respectful practice in MCH nursing.	Understand, respect, and learn from the professional experience of others.	Demonstrates routine professionalism, empathy, boundaries, collaboration, and self-regulation expected of a novice/advanced beginner.	Retained and contextualized	1.00
35 / Professional Morale	RCF PM-C3; UHC C17; CIRN 27, 30, 36; COINN; SBA/MNH; NCC MNN; NHS CCF	Working collaboratively	Professional, ethical, legal, collaborative, and respectful practice in MCH nursing.	Manage relationships with others and other staff members in a mutually respectful and collaborative manner, and resolve any conflicts that may arise.	Demonstrates routine professionalism, empathy, boundaries, collaboration, and self-regulation expected of a novice/advanced beginner.	Retained and contextualized	1.00
36 / Professional Morale	UHC C13.3/C14.3; RCF P-C3/PM-C3; CIRN 30, 32, 33; COINN; SBA/MNH; NCC MNN; NHS CCF	Working collaboratively	Professional, ethical, legal, collaborative, and respectful practice in MCH nursing.	Actively communicate and collaborate with colleagues in a timely manner regarding patient health information.	Demonstrates routine professionalism, empathy, boundaries, collaboration, and self-regulation expected of a novice/advanced beginner.	Retained and contextualized	1.00
37 / Professional Morale	UHC C6.3/C8.2; RCF PM-C4; CIRN 1, 12; COINN; SBA/MNH; NCC MNN; NHS CCF	Fulfilling professional responsibilities	Professional, ethical, legal, collaborative, and respectful practice in MCH nursing.	Flexibly adjust the order of nursing activities according to changes in clinical situations.	Demonstrates routine professionalism, empathy, boundaries, collaboration, and self-regulation expected of a novice/advanced beginner.	Retained and contextualized	1.00
38 / Professional Morale	UHC C8.1-C8.3/C16.2; RCF PM-C4; CIRN 2, 29, 35; COINN; SBA/MNH; NCC MNN; NHS CCF	Fulfilling professional responsibilities	Professional, ethical, legal, collaborative, and respectful practice in MCH nursing.	Demonstrate adaptability when facing challenges and seek support when necessary.	Demonstrates routine professionalism, empathy, boundaries, collaboration, and self-regulation expected of a novice/advanced beginner.	Retained and contextualized	1.00
39 / Professional Morale	RCF PM-A1; UHC C20.1-C20.2; ECCF PMA1; CIRN 5, 7, 41; COINN; SBA/MNH; ICNP Prenatal; NCC MNN; NHS CCF	Managing risks and hazards	Risk reporting, adverse-event response, quality improvement, and safety practices in MCH nursing services.	Report adverse events and conduct a comprehensive assessment and analysis of their causes.	Reports, participates, and cooperates in safety and quality activities under senior supervision.	Reframed for newly recruited nurse role	1.00
40 / Professional Morale	RCF PM-A1; UHC C20.1-C20.3; ECCF PMA1; CIRN 1, 41; COINN; SBA/MNH; ICNP Prenatal; NCC MNN; NHS CCF	Managing risks and hazards	Risk reporting, adverse-event response, quality improvement, and safety practices in MCH nursing services.	Actively respond to existing or potential complex and serious adverse events and nursing errors.	Reports, participates, and cooperates in safety and quality activities under senior supervision.	Reframed for newly recruited nurse role	1.00
41 / Professional Morale	RCF PM-A2; UHC C20.4; ECCF PMA2; CIRN 17, 23; COINN; SBA/MNH; NCC MNN; NHS CCF	Promoting quality improvement	Risk reporting, adverse-event response, quality improvement, and safety practices in MCH nursing services.	Participate in organizing and evaluating nursing quality improvement activities.	Reports, participates, and cooperates in safety and quality activities under senior supervision.	Retained and contextualized	1.00
42 / Learning and Development	RCF LD-C1; UHC C23; ECCF LDC1.1; CIRN 2, 29; COINN; SBA/MNH; NCC MNN; NHS CCF	Continuous learning and professional development	Continuing learning, feedback use, supervised application of new knowledge, and peer learning in MCH nursing.	Reflect in practice and seek different approaches to problem solving.	Identifies learning needs, uses feedback, participates in training, and applies new knowledge with mentor/senior support.	Retained and contextualized	1.00
43 / Learning and Development	RCF LD-C1; UHC C23; ECCF LDC1; CIRN 46; COINN; SBA/MNH; NCC MNN; NHS CCF	Continuous learning and professional development	Continuing learning, feedback use, supervised application of new knowledge, and peer learning in MCH nursing.	Actively identify one's own learning needs.	Identifies learning needs, uses feedback, participates in training, and applies new knowledge with mentor/senior support.	Retained and contextualized	1.00
44 / Learning and Development	RCF LD-C1; UHC C18.2/C23; ECCF LDC1.2; CIRN 6, 23; COINN; SBA/MNH; NCC MNN; NHS CCF	Continuous learning and professional development	Continuing learning, feedback use, supervised application of new knowledge, and peer learning in MCH nursing.	Apply new knowledge to practice with support.	Identifies learning needs, uses feedback, participates in training, and applies new knowledge with mentor/senior support.	Reframed for newly recruited nurse role	1.00
45 / Learning and	RCF LD-C1; UHC C16.4/C23; ECCF LDA1.4;	Continuous learning and professional	Continuing learning, feedback use, supervised	Adjust learning plans in a timely manner according to feedback from practice and seek	Identifies learning needs, uses feedback, participates in	Reframed for newly recruited nurse role	1.00

Item / Domain	Source basis	Extracted competency concept	MCH-related practice activity	Operationalized behaviour/task (final item wording)	Benner-informed proficiency expectation	Adaptation decision	I-CVI
Development	CIRN 29, 46, 51; COINN; SBA/MNH; NCC MNN; NHS CCF	development	application of new knowledge, and peer learning in MCH nursing.	support as needed.	training, and applies new knowledge with mentor/senior support.		
46 / Learning and Development	RCF LD-C2; ECCF LDC2; CIRN 55, 58; COINN; SBA/MNH; NCC MNN; NHS CCF	Continuous learning and professional development	Continuing learning, feedback use, supervised application of new knowledge, and peer learning in MCH nursing.	Stimulate others' learning motivation and encourage their continuous learning and development.	Identifies learning needs, uses feedback, participates in training, and applies new knowledge with mentor/senior support.	Retained and contextualized	1.00
47 / Learning and Development	RCF LD-C3/A1; ECCF LDA1; CIRN 50, 51; COINN; SBA/MNH; NCC MNN; NHS CCF	Strengthening specialized MCH nursing learning and training	Continuing learning, feedback use, supervised application of new knowledge, and peer learning in MCH nursing.	Strive to increase opportunities to participate in maternal and child health nursing education and training.	Identifies learning needs, uses feedback, participates in training, and applies new knowledge with mentor/senior support.	Retained and contextualized	1.00
48 / Learning and Development	RCF LD-C3; ECCF LDC3; CIRN 50, 56; COINN; SBA/MNH; NCC MNN; NHS CCF	Strengthening specialized MCH nursing learning and training	Continuing learning, feedback use, supervised application of new knowledge, and peer learning in MCH nursing.	Advocate for strengthening the quality and supervision of maternal and child health nursing education and training.	Identifies learning needs, uses feedback, participates in training, and applies new knowledge with mentor/senior support.	Adapted for MCH context	1.00
49 / Learning and Development	RCF LD-A1; ECCF LDA1.3; CIRN 51; COINN; SBA/MNH; NCC MNN; NHS CCF	Managing one's own professional development	Continuing learning, feedback use, supervised application of new knowledge, and peer learning in MCH nursing.	Actively participate in required education and training to achieve personal professional development goals.	Identifies learning needs, uses feedback, participates in training, and applies new knowledge with mentor/senior support.	Retained and contextualized	1.00
50 / Learning and Development	RCF LD-A1; UHC C16.2; ECCF LDA1.4; CIRN 29, 52; COINN; SBA/MNH; NCC MNN; NHS CCF	Managing one's own professional development	Continuing learning, feedback use, supervised application of new knowledge, and peer learning in MCH nursing.	Seek supervisor participation and support to meet learning and development needs.	Identifies learning needs, uses feedback, participates in training, and applies new knowledge with mentor/senior support.	Reframed for newly recruited nurse role	1.00
51 / Learning and Development	RCF LD-C2/A2; ECCF LDA2; CIRN 52, 55, 58; COINN; SBA/MNH; NCC MNN; NHS CCF	Supporting peer learning	Continuing learning, feedback use, supervised application of new knowledge, and peer learning in MCH nursing.	Encourage learning as a peer and share clinical practice experience.	Identifies learning needs, uses feedback, participates in training, and applies new knowledge with mentor/senior support.	Retained and contextualized	1.00
52 / Management and Leadership	RCF ML-C1/ML-A2; UHC C20.3; ECCF MLC1; CIRN 21, 27, 30; COINN; SBA/MNH; NCC MNN; NHS CCF	Working to improve the performance of the MCH nursing team	Team communication, service coordination, quality feedback, resource integration, and continuity across prenatal, delivery, postpartum, and child health services.	Identify communication barriers or efficiency bottlenecks in team workflows and provide constructive suggestions.	Contributes to team processes by assisting, reporting, supporting, and collecting feedback; not interpreted as independent managerial authority.	Reframed for participatory early-career role	0.93
53 / Management and Leadership	UHC C11.2/C16.1; ECCF MLC1; CIRN 20, 21; COINN; SBA/MNH; NCC MNN; NHS CCF	Working to improve the performance of the MCH nursing team	Team communication, service coordination, quality feedback, resource integration, and continuity across prenatal, delivery, postpartum, and child health services.	Actively listen to and encourage ideas and opinions from other team members.	Contributes to team processes by assisting, reporting, supporting, and collecting feedback; not interpreted as independent managerial authority.	Reframed for participatory early-career role	0.93
54 / Management and Leadership	RCF ML-C1/A1; UHC C14.3; ECCF MLC1; CIRN 30, 31; COINN; SBA/MNH; NCC MNN; NHS CCF	Working to improve the performance of the MCH nursing team	Team communication, service coordination, quality feedback, resource integration, and continuity across prenatal, delivery, postpartum, and child health services.	Participate in and cooperate with collaborative work within the MCH nursing team with support.	Contributes to team processes by assisting, reporting, supporting, and collecting feedback; not interpreted as independent managerial authority.	Reframed for participatory early-career role	0.93
55 / Management and Leadership	UHC C1.2/C2.2; ECCF PC1; RCF ML-C2; CIRN 12, 13, 16; COINN; SBA/MNH; ICNP Prenatal; NCC MNN; NHS CCF	Improving MCH service delivery capacity	Team communication, service coordination, quality feedback, resource integration, and continuity across prenatal, delivery, postpartum, and child health services.	Attend to and understand the needs and preferences of pregnant and postpartum women, children, and their family members in MCH nursing services.	Contributes to team processes by assisting, reporting, supporting, and collecting feedback; not interpreted as independent managerial authority.	Reframed for participatory early-career role	0.93
56 / Management and Leadership	RCF ML-C2; UHC C14.5/C20.3; ECCF MLC2; CIRN 17, 25, 26; COINN; SBA/MNH; NCC MNN; NHS CCF	Improving MCH service delivery capacity	Team communication, service coordination, quality feedback, resource integration, and continuity across prenatal, delivery, postpartum, and child health services.	Assist the nursing team in clarifying work goals to improve MCH nursing quality and protect the health of pregnant and postpartum women and children.	Contributes to team processes by assisting, reporting, supporting, and collecting feedback; not interpreted as independent managerial authority.	Reframed for participatory early-career role	0.93
57 / Management	RCF ML-C2/C3; ECCF MLC3; UHC C6.1; CIRN	Improving MCH service delivery capacity	Team communication, service coordination, quality	Advocate for strengthening the connection and integration of resources across MCH service	Contributes to team processes by assisting, reporting,	Reframed for participatory early-career	0.93

Item / Domain	Source basis	Extracted competency concept	MCH-related practice activity	Operationalized behaviour/task (final item wording)	Benner-informed proficiency expectation	Adaptation decision	I-CVI
and Leadership	30, 45, 48; COINN; SBA/MNH; ICNP Prenatal; NCC MNN; NHS CCF		feedback, resource integration, and continuity across prenatal, delivery, postpartum, and child health services.	processes, such as prenatal care, delivery care, postpartum rehabilitation, and child healthcare.	supporting, and collecting feedback; not interpreted as independent managerial authority.	role	
58 / Management and Leadership	RCF ML-A1/A3; ECCF MLA1/EA3; UHC C21; CIRN 22, 42, 47; COINN; SBA/MNH; NCC MNN; NHS CCF	Managing the MCH service team	Team communication, service coordination, quality feedback, resource integration, and continuity across prenatal, delivery, postpartum, and child health services.	Complete assigned tasks according to one's own abilities and scope of work, assist new colleagues in integrating into the MCH nursing team, and participate in self-evaluation and the setting of key work indicators under guidance.	Contributes to team processes by assisting, reporting, supporting, and collecting feedback; not interpreted as independent managerial authority.	Reframed for participatory early-career role	0.93
59 / Management and Leadership	RCF ML-A2; UHC C14.2-C14.3; ECCF MLA2; CIRN 22, 28, 30; COINN; SBA/MNH; NCC MNN; NHS CCF	Managing the provision of MCH nursing services	Team communication, service coordination, quality feedback, resource integration, and continuity across prenatal, delivery, postpartum, and child health services.	Assist the MCH nursing team in task allocation and implementation of responsibility mechanisms, maintain team communication mechanisms, and support information transfer.	Contributes to team processes by assisting, reporting, supporting, and collecting feedback; not interpreted as independent managerial authority.	Reframed for participatory early-career role	0.93
60 / Management and Leadership	RCF ML-A3/PM-A2; UHC C20.4; ECCF EA3/PMA2; CIRN 18, 29; COINN; SBA/MNH; ICNP Prenatal; NCC MNN; NHS CCF	Managing the provision of MCH nursing services	Team communication, service coordination, quality feedback, resource integration, and continuity across prenatal, delivery, postpartum, and child health services.	Collect and report feedback on the nursing care experience of service recipients, such as pregnant and postpartum women and children.	Contributes to team processes by assisting, reporting, supporting, and collecting feedback; not interpreted as independent managerial authority.	Reframed for participatory early-career role	0.87
61 / Management and Leadership	RCF ML-A3/PM-A2; UHC C20.4; ECCF EA3; CIRN 17, 23; COINN; SBA/MNH; NCC MNN; NHS CCF	Managing the provision of MCH nursing services	Team communication, service coordination, quality feedback, resource integration, and continuity across prenatal, delivery, postpartum, and child health services.	Assist in developing sensitive quality assessment standards for MCH nursing services with support.	Contributes to team processes by assisting, reporting, supporting, and collecting feedback; not interpreted as independent managerial authority.	Reframed for participatory early-career role	0.87
62 / Research	RCF R-C1; UHC C19.4/C18.1; ECCF EC1; CIRN 3, 8, 9; COINN; SBA/MNH; ICNP Prenatal; NCC MNN; NHS CCF	Integrating evidence into practice	Evidence-informed MCH nursing, quality improvement, documentation/data support, research participation, and dissemination.	Critically appraise research evidence with support.	Uses, discusses, collects, and disseminates evidence with support; not interpreted as independent research leadership.	Reframed as supported evidence/research participation	0.93
63 / Research	RCF R-C1; UHC C18.2; ECCF EC1; CIRN 6; COINN; SBA/MNH; ICNP Prenatal; NCC MNN; NHS CCF	Integrating evidence into practice	Evidence-informed MCH nursing, quality improvement, documentation/data support, research participation, and dissemination.	Apply research evidence to practice with support.	Uses, discusses, collects, and disseminates evidence with support; not interpreted as independent research leadership.	Reframed as supported evidence/research participation	0.93
64 / Research	UHC C12.1/C18.2; RCF R-C1; ECCF PA5/EA2; CIRN 6, 54, 57; COINN; SBA/MNH; ICNP Prenatal; NCC MNN; NHS CCF	Integrating evidence into practice	Evidence-informed MCH nursing, quality improvement, documentation/data support, research participation, and dissemination.	Inform service recipients about available research evidence.	Uses, discusses, collects, and disseminates evidence with support; not interpreted as independent research leadership.	Reframed as supported evidence/research participation	0.93
65 / Research	RCF R-C2; UHC C19.1/C18.4; ECCF EC2; CIRN 9, 23; COINN; SBA/MNH; ICNP Prenatal; NCC MNN; NHS CCF	Strengthening research evidence for MCH nursing	Evidence-informed MCH nursing, quality improvement, documentation/data support, research participation, and dissemination.	Advocate for obtaining further evidence.	Uses, discusses, collects, and disseminates evidence with support; not interpreted as independent research leadership.	Reframed as supported evidence/research participation	0.93
66 / Research	RCF R-C2/R-A1; UHC C18.4; ECCF EA1; CIRN 9, 10; COINN; SBA/MNH; ICNP Prenatal; NCC MNN; NHS CCF	Strengthening research evidence for MCH nursing	Evidence-informed MCH nursing, quality improvement, documentation/data support, research participation, and dissemination.	Initiate research with support to obtain further evidence.	Uses, discusses, collects, and disseminates evidence with support; not interpreted as independent research leadership.	Reframed as supported evidence/research participation	0.93
67 / Research	RCF R-C2; UHC C19.1-C19.3; ECCF EA3; CIRN 5, 7, 10; COINN; SBA/MNH; ICNP Prenatal; NCC MNN; NHS CCF	Strengthening research evidence for MCH nursing	Evidence-informed MCH nursing, quality improvement, documentation/data support, research participation, and dissemination.	Assist the team in assessing patient care service needs to identify research priorities.	Uses, discusses, collects, and disseminates evidence with support; not interpreted as independent research leadership.	Reframed as supported evidence/research participation	0.93
68 / Research	RCF R-A1; ECCF EA1;	Designing and	Evidence-informed MCH	Under guidance, strictly follow the research	Uses, discusses, collects, and	Reframed as supported	0.93

Item / Domain	Source basis	Extracted competency concept	MCH-related practice activity	Operationalized behaviour/task (final item wording)	Benner-informed proficiency expectation	Adaptation decision	I-CVI
	UHC C22; CIRN 10, 33, 38; COINN; SBA/MNH; ICNP Prenatal; NCC MNN; NHS CCF	conducting research	nursing, quality improvement, documentation/data support, research participation, and dissemination.	protocol and ethical principles to complete data collection and recording, and assist in data supervision, verification, and effect evaluation to ensure that evidence is authentic, complete, and traceable.	disseminates evidence with support; not interpreted as independent research leadership.	evidence/research participation	
69 / Research	RCF R-A2; ECCF EA2; CIRN 32, 33, 50; COINN; SBA/MNH; ICNP Prenatal; NCC MNN; NHS CCF	Disseminating MCH research evidence	Evidence-informed MCH nursing, quality improvement, documentation/data support, research participation, and dissemination.	Present research findings at meetings, forums, and similar venues.	Uses, discusses, collects, and disseminates evidence with support; not interpreted as independent research leadership.	Reframed as supported evidence/research participation	0.87
70 / Research	RCF R-A3; ECCF EC2/EA3; UHC C20.4; CIRN 10, 38, 50; COINN; SBA/MNH; ICNP Prenatal; NCC MNN; NHS CCF	Strengthening the research capacity of the MCH nursing team	Evidence-informed MCH nursing, quality improvement, documentation/data support, research participation, and dissemination.	Comply with relevant policies and regulations to improve research data and quality, and advocate for more funding for MCH nursing research.	Uses, discusses, collects, and disseminates evidence with support; not interpreted as independent research leadership.	Reframed as supported evidence/research participation	0.87

Note. The operationalized behaviour/task column corresponds to the final self-assessment item wording. I-CVI values were calculated as the proportion of experts rating each final item as relevant (3 or 4 on a 4-point scale); S-CVI/Ave = 0.94. Supplementary Table S2 focuses on the finalized 70 tertiary indicators after Delphi consultation and pilot testing rather than documenting every interim candidate item.

Abbreviations. UHC = WHO Global Competency and Outcomes Framework for Universal Health Coverage; RCF = WHO Rehabilitation Competency Framework; ECCF = WHO Eye Care Competency Framework; CIRN = Competency Inventory for Registered Nurses; COINN = Council of International Neonatal Nurses; SBA/MNH = WHO Defining Competent Maternal and Newborn Health Professionals / Skilled Birth Attendant and Maternal-Newborn Health professional source.; ICNP = International Classification for Nursing Practice; NCC MNN = National Certification Corporation Maternal Newborn Nursing; NHS CCF = NHS Maternity and Neonatal Core Competency Framework; MCH = maternal and child health.
