# Supplementary material for: Construction and Validation of a Contextualized Competency Framework for Newly Recruited Nurses in Maternal and Child Health Hospitals
Source: Healthcare (Basel). 2026 Jun 19;14(12):1772. doi: 10.3390/healthcare14121772 (PMC13299856; doi:10.3390/healthcare14121772)
Supplement: Supplementary file 1 [file healthcare-14-01772-s001.zip › Supplementary_Table_S3_Major_Item_Revisions_After_Pilot_Testing_Revised.pdf]

Supplementary Table S3. Major Item Revisions After Pilot Testing and Frontline User Review

Note. This table reports the 27 final questionnaire items with substantive revisions after pilot testing. Major revisions were defined as changes that affected item meaning, role level, MCH contextual specificity, self-assessment appropriateness, or alignment with the expected role boundary of newly recruited nurses. Minor grammatical polishing and routine language edits applied to other items are not listed.

Retesting note. After revision, the modified items were reviewed by the research team for clarity, developmental appropriateness, and consistency with the expected novice-to-advanced beginner role boundary before inclusion in the formal validation survey. A second independent pilot test of the revised items was not conducted and is acknowledged as a limitation in the manuscript.

Item No.	Dimension	Original wording / pre-pilot tertiary indicator	Issue identified in pilot testing	Revised wording in final questionnaire	Revision type and rationale
3	Health Advocacy and Equity	Item 3: Attend to vulnerable maternal and child health groups, such as rural women, migrant populations, and families of children with disabilities; connect them with health resources, such as free screening and charitable assistance; and promote equitable access to services.	The original wording on equity and advocacy was broad and did not clearly indicate which MCH populations required additional attention.	Item 3: I can identify the needs of vulnerable groups of women and children, such as rural women, migrant populations, and families with children with disabilities, and actively connect them with relevant health resources.	Contextualized health equity by specifying vulnerable MCH populations and resource linkage; improved observability and self-assessment suitability.
4	Health Advocacy and Equity	Item 4: Actively participate in community maternal and child health promotion activities organized by the hospital, such as reproductive health, sexually transmitted diseases, breastfeeding, and child nutrition management.	The original item could imply independent design or delivery of community MCH services, exceeding the role boundary of newly recruited nurses.	Item 4: I can assist in developing the content of community maternal and child health services with support.	Reframed as assisting with support; aligned the item with an entry-level, assisted role boundary.
5	Health Advocacy and Equity	Item 5: Actively participate in public welfare activities and community, campus, or enterprise collaborations, and proactively provide grassroots health services to improve public health literacy.	The original item could imply independent organization of public welfare or health-promotion activities.	Item 5: With support, I will participate in maternal and child health promotion activities in the community/campus.	Reframed as participation with support; emphasized participatory advocacy behavior rather than independent organization.
11	Practice	Item 11: Identify innovative solutions when addressing challenges together with patients.	The item on flexible care adjustment was abstract and lacked concrete MCH examples.	Item 11: When working with patients to address care challenges (such as children's non-cooperation with treatment and pregnant women's difficulties in post-operative recovery), I can try to flexibly adjust the plan.	Added MCH-specific examples and behavioral wording to improve contextual specificity and readability.
20	Practice	Item 20: Assess whether service recipients are at risk of harming themselves and/or others, and implement protective strategies when appropriate.	The risk-assessment item needed a clearer behavioral focus and stronger safety implications.	Item 20: I can assess whether the service recipients are at risk of self-harm or harming others, and implement corresponding protection strategies as required.	Specified self-harm or harm-to-others risk and protective strategies; made risk identification and escalation measurable.
24	Practice	Item 24: Comprehensively analyze case information, including health history, physical examination, nursing assessment, and auxiliary examinations; make nursing diagnoses; summarize case characteristics; propose nursing plans; and complete nursing records.	The item involved complex clinical reasoning and nursing diagnosis, which could be interpreted as independent advanced judgment.	Item 24: Under the guidance of a mentor, I can comprehensively analyze medical history, physical examination, nursing assessment, and auxiliary examination data, participate in nursing diagnosis, summarize case characteristics, assist in formulating nursing plans, and complete nursing records.	Added mentor guidance and participatory wording; aligned clinical reasoning expectations with novice-to-advanced beginner proficiency.
27	Practice	Item 27: Conduct follow-up after discharge and provide further nursing services when necessary.	Continuity-of-care wording was broad and needed clearer task linkage.	Item 27: I can conduct post-discharge follow-up as required by the department through telephone calls, online platforms, and other means to document the patient's condition, and assist in contacting higher-level authorities to provide further nursing services when necessary.	Specified follow-up methods, documentation, and escalation to higher-level support; linked continuity of care to actual MCH follow-up tasks.
39	Professional Morale	Item 39: Report adverse events and conduct a comprehensive assessment and analysis of their causes.	The quality and safety item could be interpreted as requiring independent handling of adverse events or complex nursing errors.	Item 39: I will promptly report adverse nursing events (e.g., extravasation of intravenous fluids, procedural errors) and collaborate with superiors to conduct cause evaluation and analysis.	Reframed as reporting, collaboration with superiors, and participation under superior guidance; preserved patient-safety responsibility while avoiding expert-level accountability.
40	Professional Morale	Item 40: Actively respond to existing or potential complex and serious adverse events and nursing errors.	The quality and safety item could be interpreted as requiring independent handling of adverse events or complex nursing errors.	Item 40: Under the guidance of superiors, I can participate in the handling of complex and major nursing adverse events and errors, and cooperate in the implementation of remedial measures.	Reframed as reporting, collaboration with superiors, and participation under superior guidance; preserved patient-safety responsibility while avoiding expert-level accountability.
46	Learning and Development	Item 46: Stimulate others' learning motivation and encourage their continuous learning and development.	Supporting others' learning could be interpreted as a formal teaching or leadership role.	Item 46: As a peer, I can consistently encourage colleagues to actively participate in learning (e.g., by sharing the value of learning and affirming their efforts), and convey the philosophy of 'active learning and continuous development' through daily communication.	Reframed as peer-level encouragement and daily communication of active-learning values.
50	Learning and Development	Item 50: Seek supervisor participation and support to meet learning and development needs.	Learning-needs clarification was too general and insufficiently observable.	Item 50: I will proactively invite superiors to hold a communication meeting to clarify one's specific needs in terms of learning and development.	Translated self-development planning into a concrete communication behavior with superiors.
54	Management and Leadership	Item 54: Participate in and cooperate with collaborative work within the MCH nursing team with support.	The item could be perceived as broad leadership or holistic-care planning rather than a behavior expected of newly recruited nurses.	Item 54: In daily nursing practice, I consistently address the multifaceted needs of service recipients, including psychological well-being, family support, and social adaptation, while proactively responding to these needs through communication and reassurance.	Operationalized the leadership-related value as direct care behavior through communication and reassurance.
56	Management and Leadership	Item 56: Assist the nursing team in clarifying work goals to improve MCH nursing quality and protect the health of pregnant and postpartum women and children.	The item on service goals or service integration could imply higher-level managerial authority.	Item 56: I can assist the nursing team in clarifying work goals for improving the quality of maternal and child care and ensuring the health of pregnant women, lying-in women, and children.	Reframed as assisting, supporting, or promoting service linkage; retained a systems perspective while limiting expectations to assisted participation.

Item No.	Dimension	Original wording / pre-pilot tertiary indicator	Issue identified in pilot testing	Revised wording in final questionnaire	Revision type and rationale
57	Management and Leadership	Item 57: Advocate for strengthening the connection and integration of resources across MCH service processes, such as prenatal care, delivery care, postpartum rehabilitation, and child healthcare.	The item on service goals or service integration could imply higher-level managerial authority.	Item 57: I will support the strengthening of the connection and resource integration in maternal and child service links such as prenatal care, childbirth care, postnatal rehabilitation, and child healthcare.	Reframed as assisting, supporting, or promoting service linkage; retained a systems perspective while limiting expectations to assisted participation.
58	Management and Leadership	Item 58: Complete assigned tasks according to one's own abilities and scope of work, assist new colleagues in integrating into the MCH nursing team, and participate in self-evaluation and the setting of key work indicators under guidance.	The item involved team management, task allocation, quality indicators, or care-experience evaluation, which may exceed the typical role of newly recruited nurses.	Item 58: I can complete the assigned tasks according to own abilities and work scope, assist new colleagues in integrating into the maternal and child care team, and participate in self-evaluation assessments and the setting of key work indicators under guidance.	Reframed using entry-level expressions such as according to my own abilities and work scope, assist, participate under guidance, collect feedback, report, and with support.
59	Management and Leadership	Item 59: Assist the MCH nursing team in task allocation and implementation of responsibility mechanisms, maintain team communication mechanisms, and support information transfer.	The item involved team management, task allocation, quality indicators, or care-experience evaluation, which may exceed the typical role of newly recruited nurses.	Item 59: I can assist the maternal and child care team in assigning tasks, implementing a responsibility mechanism, maintaining team communication mechanisms, and cooperating to complete information transmission.	Reframed using entry-level expressions such as according to my own abilities and work scope, assist, participate under guidance, collect feedback, report, and with support.
60	Management and Leadership	Item 60: Collect and report feedback on the nursing care experience of service recipients, such as pregnant and postpartum women and children.	The item involved team management, task allocation, quality indicators, or care-experience evaluation, which may exceed the typical role of newly recruited nurses.	Item 60: I can collect feedback on the care experience from service recipients such as pregnant and lying-in women and children, and report it.	Reframed using entry-level expressions such as according to my own abilities and work scope, assist, participate under guidance, collect feedback, report, and with support.
61	Management and Leadership	Item 61: Assist in developing sensitive quality assessment standards for MCH nursing services with support.	The item involved team management, task allocation, quality indicators, or care-experience evaluation, which may exceed the typical role of newly recruited nurses.	Item 61: With support, I can assist in formulating sensitive quality assessment standards for maternal and child care services.	Reframed using entry-level expressions such as according to my own abilities and work scope, assist, participate under guidance, collect feedback, report, and with support.
62	Research	Item 62: Critically appraise research evidence with support.	Evidence-based practice or evidence generation was too advanced or abstract for newly recruited nurses.	Item 62: Under the guidance of superiors or instructors, I can understand and initially judge the reference value of relevant evidence-based materials for clinical nursing.	Reframed as guided evidence-informed practice rather than independent research leadership.
63	Research	Item 63: Apply research evidence to practice with support.	Evidence-based practice or evidence generation was too advanced or abstract for newly recruited nurses.	Item 63: I can apply research evidence to practice with support.	Reframed as guided evidence-informed practice rather than independent research leadership.
64	Research	Item 64: Inform service recipients about available research evidence.	Evidence-based practice or evidence generation was too advanced or abstract for newly recruited nurses.	Item 64: I can convey relevant evidence-based information to service recipients in a popular and easy-to-understand way to help them comprehend it.	Reframed as guided evidence-informed practice rather than independent research leadership.
65	Research	Item 65: Advocate for obtaining further evidence.	Evidence-based practice or evidence generation was too advanced or abstract for newly recruited nurses.	Item 65: When encountering nursing challenges (e.g., suboptimal outcomes in special cases), I will recommend to superiors to further investigate research evidence to support decision-making.	Reframed as guided evidence-informed practice rather than independent research leadership.
66	Research	Item 66: Initiate research with support to obtain further evidence.	Evidence-based practice or evidence generation was too advanced or abstract for newly recruited nurses.	Item 66: Under guidance, I can participate in promoting the formation and improvement of further research evidence in clinical practice.	Reframed as guided evidence-informed practice rather than independent research leadership.
67	Research	Item 67: Assist the team in assessing patient care service needs to identify research priorities.	The item related to research priorities, ethical research conduct, dissemination, data quality, funding advocacy, or institutional research capacity could be interpreted as advanced research responsibility.	Item 67: I can assist the team in observing patient care service needs (e.g., gaps in rehabilitation guidance, insufficient psychological support), providing clues for identifying research priorities.	Reframed as assisting, facilitating, showcasing with support, maintaining data quality, or advocating for support within the role boundary of newly recruited nurses.
68	Research	Item 68: Under guidance, strictly follow the research protocol and ethical principles to complete data collection and recording, and assist in data supervision, verification, and effect evaluation to ensure that evidence is authentic, complete, and traceable.	The item related to research priorities, ethical research conduct, dissemination, data quality, funding advocacy, or institutional research capacity could be interpreted as advanced research responsibility.	Item 68: I can facilitate the standardized conduct and ethical compliance of research related to maternal and child health care, and provide data support for supplementing evidence-based nursing evidence.	Reframed as assisting, facilitating, showcasing with support, maintaining data quality, or advocating for support within the role boundary of newly recruited nurses.
69	Research	Item 69: Present research findings at meetings, forums, and similar venues.	The item related to research priorities, ethical research conduct, dissemination, data quality, funding advocacy, or institutional research capacity could be interpreted as advanced research responsibility.	Item 69: With support, I can showcase the research achievements in maternal and child health care through platforms such as departmental meetings and academic forums, helping to expand the dissemination scope of research content and its influence in the industry.	Reframed as assisting, facilitating, showcasing with support, maintaining data quality, or advocating for support within the role boundary of newly recruited nurses.
70	Research	Item 70: Comply with relevant policies and regulations to improve research data and quality, and advocate for more funding for MCH nursing research.	The item related to research priorities, ethical research conduct, dissemination, data quality, funding advocacy, or institutional research capacity could be interpreted as advanced research responsibility.	Item 70: I can abide by relevant policies and regulations to ensure the quality of research data, while advocating for more funding for maternal and child care research and promoting the improvement of the research capabilities of maternal and child care institutions.	Reframed as assisting, facilitating, showcasing with support, maintaining data quality, or advocating for support within the role boundary of newly recruited nurses.

Source note. The final questionnaire wording was compared with the pre-pilot tertiary indicators. This supplementary table focuses on substantive revisions that affected meaning, role level, contextual specificity, or self-assessment appropriateness.
