# Supplementary material for: Construction and Validation of a Contextualized Competency Framework for Newly Recruited Nurses in Maternal and Child Health Hospitals
Source: Healthcare (Basel). 2026 Jun 19;14(12):1772. doi: 10.3390/healthcare14121772 (PMC13299856; doi:10.3390/healthcare14121772)
Supplement: Supplementary file 1 [file healthcare-14-01772-s001.zip › Supplementary_Table_S4_Item_Level_Analysis_with_Descriptives.pdf]

Supplementary Table S4. Item-Level Analysis of the Final 70-Item NCF-MCH

Note. This table integrates item descriptives (mean, standard deviation, and coefficient of variation), item-total correlations, item discrimination, corrected item-total correlations, and Cronbach's alpha if item deleted for the final 70-item Nursing Competency Framework for newly recruited nurses in Maternal and Child Health Hospitals (NCF-MCH). All item-total correlations were statistically significant (p = 0.001), and all critical ratio values were statistically significant (p < 0.001). Items with corrected item-total correlations below 0.30 are flagged for cautious interpretation. Item retention should be interpreted as preserving initial content coverage and contextual relevance, not as evidence that all items are equally necessary.

Indicator	Result / interpretation
Sample size	n = 554
Mean score	3.45 to 4.16
Standard deviation	0.91 to 1.11
Coefficient of variation (CV)	0.22 to 0.30
Item-total correlation with total score	0.261 to 0.674
Critical ratio (CR)	3.452 to 16.350
Corrected item-total correlation	0.233 to 0.656
Cronbach's alpha if item deleted	0.942 to 0.944
Items with corrected item-total correlation < 0.30	Q1, Q3, Q4, Q40, Q52, Q53, Q62, Q63, Q64
Interpretation	Most items showed acceptable discrimination; several items require cautious interpretation and further testing for possible refinement or short-form development.

Detailed item-level results

Item	Mean	SD	CV	Item-total r	p	CR	Corrected item-total r	Alpha if item deleted	Review note
Q1	3.88	0.94	0.24	0.276	0.001	5.859	0.248	0.944	Flagged: corrected item-total correlation < 0.30
Q2	3.93	0.95	0.24	0.348	0.001	7.555	0.321	0.944	Retained; acceptable basic item performance
Q3	3.78	1.00	0.26	0.311	0.001	6.323	0.282	0.944	Flagged: corrected item-total correlation < 0.30
Q4	3.87	0.99	0.26	0.294	0.001	5.449	0.265	0.944	Flagged: corrected item-total correlation < 0.30
Q5	3.87	1.01	0.26	0.375	0.001	8.384	0.347	0.944	Retained; acceptable basic item performance
Q6	3.97	1.03	0.26	0.594	0.001	12.627	0.572	0.943	Retained; acceptable basic item performance
Q7	3.90	1.02	0.26	0.591	0.001	13.103	0.569	0.943	Retained; acceptable basic item performance
Q8	3.96	0.99	0.25	0.572	0.001	10.867	0.551	0.943	Retained; acceptable basic item performance
Q9	4.14	0.91	0.22	0.463	0.001	9.683	0.440	0.943	Retained; acceptable basic item performance
Q10	3.79	0.99	0.26	0.674	0.001	16.350	0.656	0.942	Retained; acceptable basic item performance
Q11	3.73	0.98	0.26	0.563	0.001	13.369	0.541	0.943	Retained; acceptable basic item performance
Q12	3.99	1.00	0.25	0.594	0.001	12.491	0.573	0.943	Retained; acceptable basic item performance
Q13	4.09	0.98	0.24	0.583	0.001	10.689	0.562	0.943	Retained; acceptable basic item performance
Q14	3.88	1.01	0.26	0.578	0.001	12.174	0.556	0.943	Retained; acceptable basic item performance
Q15	3.93	0.98	0.25	0.567	0.001	10.608	0.545	0.943	Retained; acceptable basic item performance
Q16	3.95	1.05	0.26	0.645	0.001	14.792	0.625	0.943	Retained; acceptable basic item performance
Q17	3.93	1.01	0.26	0.584	0.001	11.787	0.562	0.943	Retained; acceptable basic item performance
Q18	3.88	1.00	0.26	0.593	0.001	12.642	0.572	0.943	Retained; acceptable basic item performance
Q19	3.79	0.97	0.26	0.574	0.001	12.063	0.552	0.943	Retained; acceptable basic item performance
Q20	3.91	1.03	0.26	0.631	0.001	13.539	0.611	0.943	Retained; acceptable basic item performance
Q21	3.83	0.99	0.26	0.598	0.001	12.642	0.577	0.943	Retained; acceptable basic item performance
Q22	4.08	0.98	0.24	0.549	0.001	10.206	0.526	0.943	Retained; acceptable basic item performance
Q23	3.90	0.98	0.25	0.643	0.001	13.793	0.624	0.943	Retained; acceptable basic item performance
Q24	3.84	0.99	0.26	0.579	0.001	12.947	0.557	0.943	Retained; acceptable basic item performance
Q25	3.84	1.00	0.26	0.591	0.001	12.083	0.570	0.943	Retained; acceptable basic item performance
Q26	3.91	0.99	0.25	0.568	0.001	10.949	0.546	0.943	Retained; acceptable basic item performance
Q27	3.75	1.00	0.27	0.579	0.001	12.927	0.557	0.943	Retained; acceptable basic item performance
Q28	3.90	0.93	0.24	0.512	0.001	11.727	0.490	0.943	Retained; acceptable basic item performance
Q29	3.95	1.00	0.25	0.522	0.001	13.053	0.498	0.943	Retained; acceptable basic item performance
Q30	3.94	0.98	0.25	0.470	0.001	11.925	0.445	0.943	Retained; acceptable basic item performance
Q31	4.05	0.98	0.24	0.519	0.001	12.074	0.496	0.943	Retained; acceptable basic item performance
Q32	4.12	0.95	0.23	0.462	0.001	10.341	0.438	0.943	Retained; acceptable basic item performance
Q33	4.00	0.98	0.25	0.551	0.001	14.050	0.528	0.943	Retained; acceptable basic item performance
Q34	4.14	0.93	0.22	0.439	0.001	9.760	0.414	0.943	Retained; acceptable basic item performance
Q35	3.96	0.95	0.24	0.498	0.001	13.134	0.475	0.943	Retained; acceptable basic item performance
Q36	3.81	0.97	0.25	0.525	0.001	13.113	0.502	0.943	Retained; acceptable basic item performance
Q37	3.98	0.96	0.24	0.481	0.001	11.922	0.457	0.943	Retained; acceptable basic item performance
Q38	3.89	1.00	0.26	0.410	0.001	8.678	0.383	0.944	Retained; acceptable basic item performance
Q39	3.92	1.00	0.25	0.361	0.001	8.295	0.333	0.944	Retained; acceptable basic item performance
Q40	3.90	0.95	0.24	0.323	0.001	7.079	0.295	0.944	Flagged: corrected item-total correlation < 0.30
Q41	3.88	0.96	0.25	0.355	0.001	8.082	0.328	0.944	Retained; acceptable basic item performance
Q42	4.00	1.00	0.25	0.343	0.001	6.377	0.315	0.944	Retained; acceptable basic item performance
Q43	3.92	0.95	0.24	0.440	0.001	9.817	0.415	0.943	Retained; acceptable basic item performance
Q44	4.06	0.93	0.23	0.393	0.001	8.083	0.367	0.944	Retained; acceptable basic item performance
Q45	3.98	0.95	0.24	0.353	0.001	6.478	0.326	0.944	Retained; acceptable basic item performance
Q46	3.85	1.00	0.26	0.417	0.001	9.595	0.390	0.944	Retained; acceptable basic item performance
Q47	3.95	0.95	0.24	0.443	0.001	9.763	0.418	0.943	Retained; acceptable basic item performance

Item	Mean	SD	CV	Item-total r	p	CR	Corrected item-total r	Alpha if item deleted	Review note
Q48	3.87	1.00	0.26	0.490	0.001	11.269	0.466	0.943	Retained; acceptable basic item performance
Q49	3.91	1.01	0.26	0.428	0.001	8.656	0.401	0.944	Retained; acceptable basic item performance
Q50	3.79	0.99	0.26	0.412	0.001	9.597	0.386	0.944	Retained; acceptable basic item performance
Q51	3.90	0.95	0.24	0.409	0.001	8.535	0.383	0.944	Retained; acceptable basic item performance
Q52	4.09	1.01	0.25	0.267	0.001	3.452	0.237	0.944	Flagged: corrected item-total correlation < 0.30
Q53	4.16	0.93	0.22	0.261	0.001	4.251	0.233	0.944	Flagged: corrected item-total correlation < 0.30
Q54	3.84	1.01	0.26	0.427	0.001	8.702	0.400	0.944	Retained; acceptable basic item performance
Q55	3.87	0.99	0.26	0.421	0.001	9.528	0.395	0.944	Retained; acceptable basic item performance
Q56	3.83	0.94	0.25	0.448	0.001	10.770	0.424	0.943	Retained; acceptable basic item performance
Q57	3.96	0.98	0.25	0.386	0.001	7.412	0.359	0.944	Retained; acceptable basic item performance
Q58	3.81	0.97	0.25	0.427	0.001	9.135	0.401	0.944	Retained; acceptable basic item performance
Q59	4.06	1.03	0.25	0.370	0.001	6.512	0.342	0.944	Retained; acceptable basic item performance
Q60	3.88	0.97	0.25	0.408	0.001	8.426	0.382	0.944	Retained; acceptable basic item performance
Q61	3.89	0.99	0.26	0.356	0.001	6.996	0.328	0.944	Retained; acceptable basic item performance
Q62	3.80	1.05	0.28	0.311	0.001	5.622	0.280	0.944	Flagged: corrected item-total correlation < 0.30
Q63	3.84	1.03	0.27	0.269	0.001	4.709	0.238	0.944	Flagged: corrected item-total correlation < 0.30
Q64	3.73	1.11	0.30	0.315	0.001	5.817	0.283	0.944	Flagged: corrected item-total correlation < 0.30
Q65	3.76	1.08	0.29	0.345	0.001	7.869	0.315	0.944	Retained; acceptable basic item performance
Q66	3.56	1.05	0.29	0.362	0.001	7.736	0.332	0.944	Retained; acceptable basic item performance
Q67	3.65	1.03	0.28	0.361	0.001	6.926	0.332	0.944	Retained; acceptable basic item performance
Q68	3.68	1.08	0.29	0.347	0.001	7.574	0.316	0.944	Retained; acceptable basic item performance
Q69	3.45	1.04	0.30	0.341	0.001	7.320	0.311	0.944	Retained; acceptable basic item performance
Q70	3.50	1.05	0.30	0.337	0.001	8.365	0.307	0.944	Retained; acceptable basic item performance

Interpretive note. All 70 items met the basic item discrimination criterion based on the CR values. The descriptive statistics showed that item means ranged from 3.45 to 4.16, standard deviations ranged from 0.91 to 1.11, and coefficients of variation ranged from 0.22 to 0.30. However, several items had corrected item-total correlations below 0.30, suggesting that they should be reviewed in future validation studies. Because the present study aimed to retain a comprehensive context-specific framework, item retention was based on statistical performance together with theoretical relevance, MCH contextual importance, and Delphi expert feedback. Future studies should further examine redundancy, ceiling effects, and item reduction using larger samples and additional psychometric methods.
