# Supplementary material for: Construction and Validation of a Contextualized Competency Framework for Newly Recruited Nurses in Maternal and Child Health Hospitals
Source: Healthcare (Basel). 2026 Jun 19;14(12):1772. doi: 10.3390/healthcare14121772 (PMC13299856; doi:10.3390/healthcare14121772)
Supplement: Supplementary file 1 [file healthcare-14-01772-s001.zip › Supplementary_Table_S5_Rotated_Pattern_Matrix_EFA.pdf]

Supplementary Table S5. Rotated Pattern Matrix from Exploratory Factor Analysis

Note. Extraction method: principal axis factoring. Rotation method: Promax with Kaiser normalization. The pattern matrix is reported because an oblique rotation was used. Primary loadings are shown in bold. No secondary loading greater than or equal to 0.30 was observed in the displayed matrix; therefore, no substantial cross-loading was identified based on this criterion.

Interpretive caution. The six-factor solution corresponded closely to the theoretically developed item blocks. This pattern should be interpreted as preliminary structural evidence rather than definitive confirmation, because it may also reflect conceptual preassignment, wording similarity within domains, and the original questionnaire organization. Factor 1 contained 22 items and should be further examined for possible subdomains or item reduction in future studies.

Item	Factor 1	Factor 2	Factor 3	Factor 4	Factor 5	Factor 6	Cross-loading >= .30
Q12	.802	-.101	.078	.045	-.021	-.026	No
Q13	.799	-.003	.015	-.025	-.086	.028	No
Q15	.785	-.081	.039	.046	-.075	-.067	No
Q22	.784	-.157	.012	.007	-.045	.070	No
Q17	.781	.000	.028	-.006	-.013	-.076	No
Q8	.778	-.058	-.007	-.021	.025	-.007	No
Q16	.777	.047	.030	.036	-.077	-.013	No
Q18	.767	.066	-.099	-.077	-.007	.017	No
Q14	.755	.000	.033	.028	.054	-.074	No
Q19	.750	-.013	.103	-.085	.022	-.001	No
Q7	.743	-.032	-.069	-.019	.083	-.017	No
Q24	.741	-.043	.008	-.034	.018	-.017	No
Q26	.740	.064	-.020	.023	-.018	-.125	No
Q23	.739	.008	.128	.010	.059	.030	No
Q25	.734	-.041	.007	.052	-.073	.068	No
Q10	.724	.095	-.029	.069	.050	.042	No

Item	Factor 1	Factor 2	Factor 3	Factor 4	Factor 5	Factor 6	Cross-loading >= .30
Q21	.722	.039	-.025	-.004	.038	.099	No
Q11	.722	.106	-.083	-.030	.011	-.054	No
Q20	.719	.042	-.002	.034	.108	-.006	No
Q6	.711	.141	.000	-.083	.009	.060	No
Q9	.710	-.074	-.135	-.029	-.095	.039	No
Q27	.688	-.007	-.026	.044	.057	.058	No
Q34	-.041	.794	.037	.044	-.099	-.058	No
Q32	-.096	.788	.039	.051	-.066	.022	No
Q37	-.003	.762	.010	.016	.106	-.042	No
Q41	-.046	.756	-.050	-.069	-.077	-.041	No
Q29	.082	.752	.002	.046	-.053	.026	No
Q35	.053	.747	-.009	-.019	.025	.013	No
Q31	.035	.741	.004	.090	-.077	-.029	No
Q33	.084	.738	.033	-.025	.016	.020	No
Q38	-.049	.736	.017	-.056	.011	-.053	No
Q28	-.001	.712	-.011	.068	.075	.043	No
Q30	-.028	.703	-.040	.002	.003	.148	No
Q36	.022	.701	.058	.044	.005	.025	No
Q40	-.052	.658	-.005	-.136	.056	-.119	No

Item	Factor 1	Factor 2	Factor 3	Factor 4	Factor 5	Factor 6	Cross-loading >= .30
Q39	-.002	.640	-.073	-.053	.086	.028	No
Q53	.008	-.095	.856	-.030	-.001	-.125	No
Q59	-.012	-.044	.845	-.002	.061	-.003	No
Q52	-.132	-.041	.817	-.025	-.009	.039	No
Q61	-.040	-.018	.816	-.050	.034	.033	No
Q57	.008	.030	.816	-.008	-.052	.009	No
Q54	-.016	.060	.796	.005	.027	-.005	No
Q56	-.019	.020	.796	.045	.005	.046	No
Q58	.026	.045	.767	.014	.010	.034	No
Q55	.084	.084	.756	-.051	-.003	-.006	No
Q60	.099	-.021	.729	.054	-.060	-.008	No
Q44	.045	-.083	-.086	.835	-.005	-.030	No
Q42	-.063	.022	-.026	.819	-.010	-.105	No
Q49	-.029	-.084	.104	.785	.021	.034	No
Q47	.020	.056	-.040	.763	.020	-.033	No
Q48	.093	.009	.047	.761	-.006	-.038	No
Q46	-.024	.035	-.101	.747	.021	.042	No
Q43	-.035	.035	.072	.744	-.126	.070	No
Q45	.003	-.078	-.039	.738	-.001	.036	No

Item	Factor 1	Factor 2	Factor 3	Factor 4	Factor 5	Factor 6	Cross-loading >= .30
Q51	-.075	.056	.032	.726	.075	.022	No
Q50	.041	.017	.006	.668	.047	-.006	No
Q62	.010	-.027	.007	-.005	.831	-.047	No
Q63	-.014	-.054	-.102	.004	.801	-.011	No
Q65	.000	-.020	-.002	.045	.801	-.003	No
Q64	-.049	-.013	-.040	-.021	.780	.088	No
Q66	.098	.065	-.019	-.024	.770	-.037	No
Q70	-.045	-.002	.075	.011	.769	-.020	No
Q69	.011	-.017	.066	.045	.759	-.021	No
Q67	-.030	.027	.083	-.006	.734	.047	No
Q68	.020	.051	-.052	-.013	.730	.018	No
Q4	-.008	-.013	-.061	-.026	-.004	.802	No
Q2	-.019	.064	.036	-.042	-.031	.794	No
Q5	.053	.045	-.006	.017	-.040	.764	No
Q3	-.017	-.060	.060	.043	.063	.757	No
Q1	-.002	-.075	-.009	-.009	.022	.736	No

Abbreviations. EFA = exploratory factor analysis. Blank cells indicate loadings suppressed in the SPSS output.
