# Supplementary material for: Construction and Validation of a Contextualized Competency Framework for Newly Recruited Nurses in Maternal and Child Health Hospitals
Source: Healthcare (Basel). 2026 Jun 19;14(12):1772. doi: 10.3390/healthcare14121772 (PMC13299856; doi:10.3390/healthcare14121772)
Supplement: Supplementary file 1 [file healthcare-14-01772-s001.zip › Supplementary_Table_S6_CFA_Standardized_Factor_Loadings.pdf]

Supplementary Table S6. Standardized Factor Loadings From Confirmatory Factor Analysis

Sample: n = 226. Estimates are standardized factor loadings from the CFA model. AVE and CR are domain-level indices and are shown in the first row of each domain.

Domain	Item No.	Path	Standardized factor loading	AVE	CR
Learning and Development	Q42	Q42 <--- LD	0.780	0.615	0.941
Learning and Development	Q43	Q43 <--- LD	0.806		
Learning and Development	Q44	Q44 <--- LD	0.800		
Learning and Development	Q45	Q45 <--- LD	0.791		
Learning and Development	Q46	Q46 <--- LD	0.800		
Learning and Development	Q47	Q47 <--- LD	0.772		
Learning and Development	Q48	Q48 <--- LD	0.827		
Learning and Development	Q49	Q49 <--- LD	0.764		
Learning and Development	Q50	Q50 <--- LD	0.732		
Learning and Development	Q51	Q51 <--- LD	0.766		
Health Advocacy and Equity	Q1	Q1 <--- HAE	0.803	0.578	0.872
Health Advocacy and Equity	Q2	Q2 <--- HAE	0.795		
Health Advocacy and Equity	Q3	Q3 <--- HAE	0.704		
Health Advocacy and Equity	Q4	Q4 <--- HAE	0.757		
Health Advocacy and Equity	Q5	Q5 <--- HAE	0.737		
Practice	Q6	Q6 <--- P	0.772	0.601	0.971
Practice	Q7	Q7 <--- P	0.770		
Practice	Q8	Q8 <--- P	0.790		
Practice	Q9	Q9 <--- P	0.751		
Practice	Q10	Q10 <--- P	0.759		
Practice	Q11	Q11 <--- P	0.744		
Practice	Q12	Q12 <--- P	0.779		
Practice	Q13	Q13 <--- P	0.767		
Practice	Q14	Q14 <--- P	0.788		
Practice	Q15	Q15 <--- P	0.817		
Practice	Q16	Q16 <--- P	0.789		
Practice	Q17	Q17 <--- P	0.762		
Practice	Q18	Q18 <--- P	0.802		
Practice	Q19	Q19 <--- P	0.735		
Practice	Q20	Q20 <--- P	0.776		
Practice	Q21	Q21 <--- P	0.748		
Practice	Q22	Q22 <--- P	0.797		
Practice	Q23	Q23 <--- P	0.796		
Practice	Q24	Q24 <--- P	0.789		
Practice	Q25	Q25 <--- P	0.814		
Practice	Q26	Q26 <--- P	0.768		
Practice	Q27	Q27 <--- P	0.729		
Professional Morale	Q41	Q41 <--- PM	0.679	0.527	0.939
Professional Morale	Q40	Q40 <--- PM	0.596		
Professional Morale	Q39	Q39 <--- PM	0.581		

Domain	Item No.	Path	Standardized factor loading	AVE	CR
Professional Morale	Q38	Q38 <--- PM	0.721		
Professional Morale	Q37	Q37 <--- PM	0.770		
Professional Morale	Q36	Q36 <--- PM	0.809		
Professional Morale	Q35	Q35 <--- PM	0.707		
Professional Morale	Q34	Q34 <--- PM	0.768		
Professional Morale	Q33	Q33 <--- PM	0.776		
Professional Morale	Q32	Q32 <--- PM	0.717		
Professional Morale	Q31	Q31 <--- PM	0.768		
Professional Morale	Q30	Q30 <--- PM	0.742		
Professional Morale	Q29	Q29 <--- PM	0.776		
Professional Morale	Q28	Q28 <--- PM	0.708		
Research	Q70	Q70 <--- R	0.779	0.642	0.942
Research	Q69	Q69 <--- R	0.757		
Research	Q68	Q68 <--- R	0.775		
Research	Q67	Q67 <--- R	0.784		
Research	Q66	Q66 <--- R	0.804		
Research	Q65	Q65 <--- R	0.804		
Research	Q64	Q64 <--- R	0.850		
Research	Q63	Q63 <--- R	0.832		
Research	Q62	Q62 <--- R	0.819		
Management and Leadership	Q60	Q60 <--- ML	0.707	0.519	0.915
Management and Leadership	Q59	Q59 <--- ML	0.721		
Management and Leadership	Q58	Q58 <--- ML	0.708		
Management and Leadership	Q57	Q57 <--- ML	0.766		
Management and Leadership	Q56	Q56 <--- ML	0.661		
Management and Leadership	Q55	Q55 <--- ML	0.748		
Management and Leadership	Q54	Q54 <--- ML	0.751		
Management and Leadership	Q53	Q53 <--- ML	0.737		
Management and Leadership	Q52	Q52 <--- ML	0.701		
Management and Leadership	Q61	Q61 <--- ML	0.695		

Note:LD=Learning and Development; ML=Management and Leadership; R=Research; PM=Professional Morale; P=Practice; HAE=Health Advocacy and Equity; CR: composite reliability; AVE: average variance extracted values.
